# Cell death-inducing cytotoxicity in truncated KCNQ4 variants associated with DFNA2 hearing loss

**DOI:** 10.1242/dmm.049015

**Published:** 2021-11-26

**Authors:** Takashi Kojima, Koichiro Wasano, Satoe Takahashi, Kazuaki Homma

**Affiliations:** 1 Department of Otolaryngology – Head and Neck Surgery, Feinberg School of Medicine, Northwestern University, Chicago, IL 60611, USA; 2 Department of Otolaryngology, Head and Neck Surgery, Keio University School of Medicine, 35 Shinanomachi, Shinjuku, Tokyo 160-8582, Japan; 3 Laboratory of Auditory Disorders, Division of Hearing and Balance Research, National Institute of Sensory Organs, National Hospital Organization Tokyo Medical Center, 2-5-1 Higashigaoka, Meguro, Tokyo 152-8902, Japan; 4 The Hugh Knowles Center for Clinical and Basic Science in Hearing and Its Disorders, Northwestern University, Evanston, IL 60608, USA

**Keywords:** KCNQ family, KCNQ4, Kv7.4, Potassium channel, Hereditary hearing loss, DFNA2

## Abstract

*KCNQ4* encodes the homotetrameric voltage-dependent potassium ion channel Kv7.4, and is the causative gene for autosomal dominant nonsyndromic sensorineural hearing loss, DFNA2. Dominant-negative inhibition accounts for the observed dominant inheritance of many DFNA2-associated *KCNQ4* variants. In addition, haploinsufficiency has been presumed as the pathological mechanism for truncated Kv7.4 variants lacking the C-terminal tetramerization region, as they are unlikely to exert a dominant-negative inhibitory effect. Such truncated Kv7.4 variants should result in relatively mild hearing loss when heterozygous; however, this is not always the case. In this study, we characterized Kv7.4^Q71fs^ (c.211delC), Kv7.4^W242X^ (c.725G>A) and Kv7.4^A349fs^ (c.1044_1051del8) in heterologous expression systems and found that expression of these truncated Kv7.4 variants induced cell death. We also found similar cell death-inducing cytotoxic effects in truncated Kv7.1 (KCNQ1) variants, suggesting that the generality of our findings could account for the dominant inheritance of many, if not most, truncated Kv7 variants. Moreover, we found that the application of autophagy inducers can ameliorate the cytotoxicity, providing a novel insight for the development of alternative therapeutic strategies for Kv7.4 variants.

## Abstract (precis)

**Summary:** Expression of truncated KCNQ4 variants lacking the C-terminal tetramerization domain results in cell-death inducing cytotoxicity, providing novel insight into the development of alternative therapeutic strategies for DFNA2 hearing loss.

## INTRODUCTION

The homotetrameric voltage-dependent potassium ion channel Kv7.4 is encoded by *KCNQ4*, and is abundantly expressed in the cochlear outer hair cells (OHCs). The potassium ion conductance mediated by Kv7.4 contributes to the establishment of a normal resting membrane electric potential (V_res_) and is crucial for repolarizing the cells after sound-elicited OHC depolarization. The large Kv7.4-mediated conductance in OHCs also contributes to reduce the membrane time constant so that the receptor potential-induced mechanical response of OHCs, i.e. electromotility (Brownell et al., 1985; Evans and Dallos, 1993), can keep up with the sound stimuli at high frequencies (Johnson et al., 2011). Although *KCNQ4* is also expressed in other cell types, OHC is the most vulnerable cell type severely affected by pathogenic *KCNQ4* variants in the cochlea. As such, dysfunction and subsequent degeneration of OHCs underlie autosomal dominant progressive sensorineural hearing loss caused by pathogenic *KCNQ4* variants (DFNA2) (Kharkovets et al., 2006). A recent study also reported a later-onset degeneration of the inner hair cells and the spiral ganglion neurons (Carignano et al., 2019). Currently, more than 50 *KCNQ4* variants associated with DFNA2 have been identified (Human Gene Mutation Database, August 2021; Stenson et al., 2017). DFNA2 hearing loss is typically more prominent at higher frequencies, although middle and low frequencies are also affected later in life (Kubisch et al., 1999). The severity of hearing loss and the rate of progression vary among *KCNQ4* variants. It is considered that the dominant inheritance of *KCNQ4* variants is ascribed to either dominant-negative inhibition or haploinsufficiency (Maljevic et al., 2010). Truncated Kv7.4 variants lacking the C-terminal tetramerization region are unlikely to form multimers with the wild-type (WT) subunit and, thus, are predicted not to exert a dominant-negative inhibitory effect. Therefore, they are thought to cause relatively mild hearing loss due to haploinsufficiency. The slowly progressive hearing loss found in patients with the heterozygous Kv7.4^Q71fs^ (c.211delC) truncation variant (Kamada et al., 2006) is in line with such a view. However, Kv7.4^W242X^ (c.725G>A) is associated with severe to profound hearing loss despite the complete lack of the pore-forming region and the subsequent C-terminal cytosolic domain (Hildebrand et al., 2008). Incidentally, heterozygous *Kcnq4* knockout mice (*Kcnq4^+/−^*) do not suffer from hearing loss (Kharkovets et al., 2006), suggesting that one functional *Kcnq4* allele is sufficient for maintaining OHCs and their normal function. The presence of a recessively inherited truncated variant, Kv7.4^A349fs^ (c.1044_1051del8) (Wasano et al., 2015), is also incompatible with a haploinsufficiency-based pathological mechanism in DFNA2 hearing loss.

In the present study, we thoroughly characterized the three truncated Kv7.4 variants, Kv7.4^Q71fs^, Kv7.4^W242X^ and Kv7.4^A349fs^, in a heterologous expression system to examine their pathological roles. We found that these Kv7.4 variants lacking the C-terminal tetramerization region are nonfunctional by themselves and incapable of forming a heteromultimer with Kv7.4^WT^. Unexpectedly, we found that the expression of these truncated Kv7.4 variants severely affected cell viability and induced cell death. We also found similar cytotoxic effects in several truncated Kv7.1 (*KCNQ1*) variants, indicating the presence of many other cytotoxic *KCNQ* variants. Moreover, we found that the cytotoxic effects of these variants could be ameliorated by the application of small molecules that modulate autophagy and/or apoptosis pathways. Collectively, our results identify cytotoxicity as a possible disease mechanism for some Kv7.4 variants, and provide a novel insight into the development of an alternative pharmacological strategy that complements current efforts to benefit patients.

## RESULTS

### Kv7.4^Q71fs^, Kv7.4^W242X^ and Kv7.4^A349fs^ do not form a functional ion channel

**Fig. 1.**
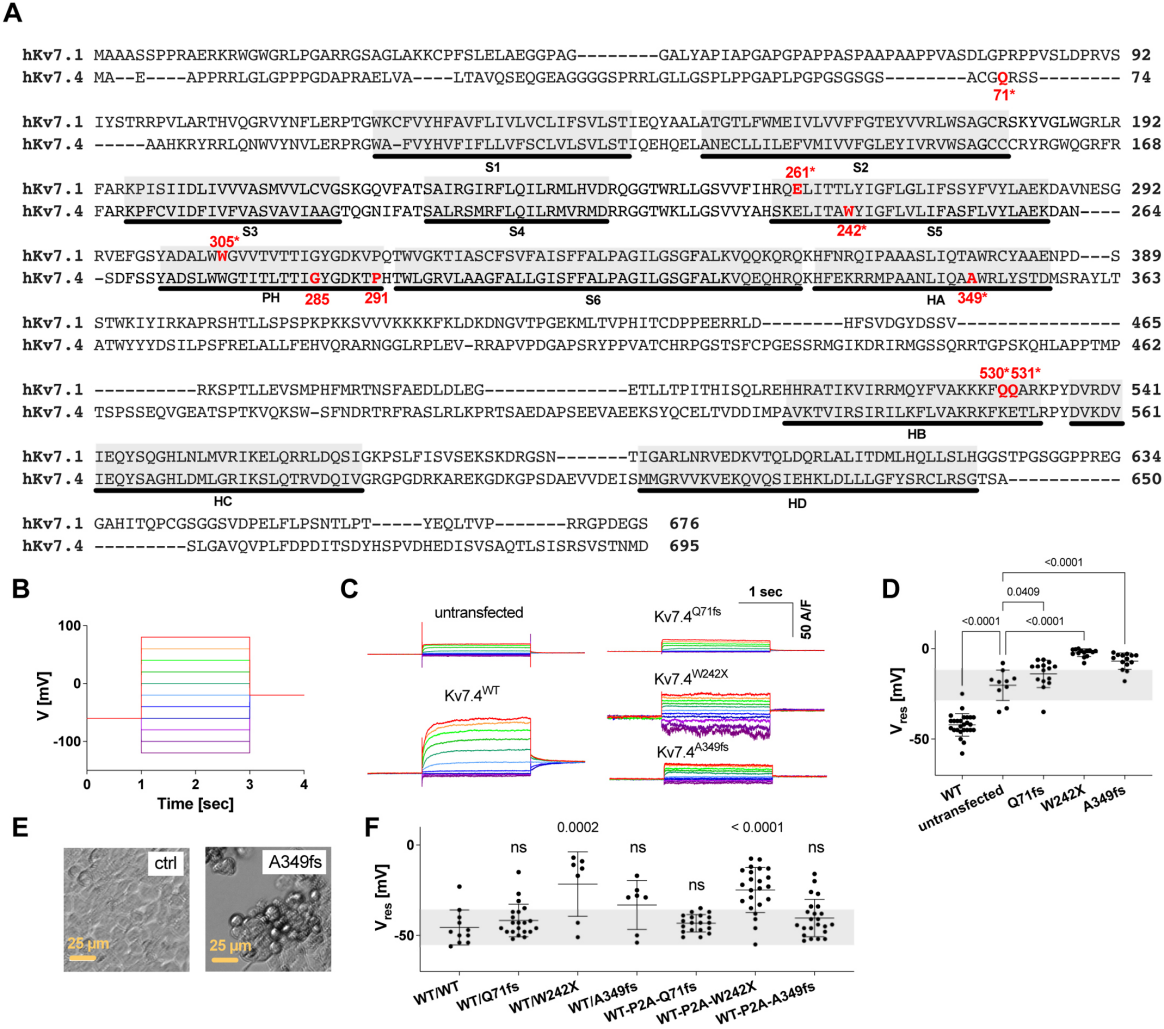
**Electrophysiological functional characterization of Kv7.4 constructs.** (A) The amino acid sequences of human Kv7.1 and human Kv7.4. Gray highlights indicate α-helices. All Kv channels possess six transmembrane (TM) segments (S1-S6). The first four TM segments (S1-S4) constitute the voltage sensing domain, and the last two, which flank a channel pore loop (P-loop), constitute the pore-forming domain (S5-PH-S6). Tetramerization of Kv7 channels is mediated by the C-terminal cytosolic helices HC and HD. The residues referred to in the main text are shown in red. An asterisk indicates a nonsense or frameshifting change that results in the truncation of the Kv7.1 or Kv7.4 protein. (B) The step voltage commands used for the voltage-clamp recordings. (C) Examples of current responses to the voltage commands shown in B. (D) A summary of the resting membrane potentials of cells expressing the Kv7.4 constructs, which were directly measured by whole-cell current-clamp (at 0 nA). Data are mean±s.d. The horizontal gray highlight indicates the mean±s.d. determined for untransfected (ctrl) cells. Adjusted *P*-values shown in the panel were determined by ANOVA followed by the Tukey–Kramer multiple comparison test. (E) Images of untransfected (ctrl) and Kv7.4^A349fs^-expressing HEK293T cells. (F) A summary of the resting membrane potentials of cells co-expressing WT and truncated human Kv7.4 variants. ANOVA followed by the Tukey–Kramer multiple comparison test was performed to obtain adjusted *P* values (in comparison to WT/WT). Data are mean±s.d. The horizontal gray highlight indicates the mean±s.d. determined for WT/WT. ns, not significant.

Four Kv7 subunits need to multimerize to complete a functional potassium ion channel (Sun and MacKinnon, 2017, 2020). Tetramerization of the Kv7 subunits is mediated by the C-terminal α-helices C and D (HC and HD in Fig. 1A) (Howard et al., 2007; Sachyani et al., 2014; Schwake et al., 2006; Sun and MacKinnon, 2017, 2020; Wiener et al., 2008; Xu et al., 2013; Xu and Minor, 2009; Li et al., 2021). Kv7.4^Q71fs^, Kv7.4^W242X^ and Kv7.4^A349fs^ lack the C-terminal cytosolic domain containing these tetramerization helices (Fig. 1A). Therefore, these variants do not likely exert a dominant-negative inhibitory effect on the Kv7.4^WT^ subunit, and, hence, they are predicted to result in less severe hearing loss when heterozygous. In fact, hearing loss in patients with heterozygous Kv7.4^Q71fs^ is relatively mild (Kamada et al., 2006). However, patients with heterozygous Kv7.4^W242X^ suffer severe to profound hearing loss (Hildebrand et al., 2008). Interestingly, patients with heterozygous Kv7.4^A349fs^ do not suffer from hearing loss, i.e. this truncated Kv7.4 variant is inherited recessively (Wasano et al., 2015). To understand the cause of these phenotypic differences, we performed whole-cell patch-clamp recordings in HEK293T cells that singly expressed Kv7.4^Q71fs^, Kv7.4^W242X^ or Kv7.4^A349fs^, or co-expressed each of these truncated variants and Kv7.4^WT^ (Fig. 1B-F) in a doxycycline-dependent manner. As functional expression of Kv7.4 greatly hyperpolarizes the resting membrane potential (V_res_) of cells, we used the V_res_ values as the metric of Kv7.4-mediated ion channel activity and compared this among cell lines (Fig. 1D-F). As expected, the expression of these truncated Kv7.4 variants did not confer potassium ion channel activity on HEK293T cells (Fig. 1C). Consistently, hyperpolarization of V_res_ (with respect to untransfected cells) was not observed for any of these variants (Fig. 1D), suggesting that these truncated Kv7.4 variants do not form a functional channel by themselves. Notably, we observed rounding and subsequent detachment of Kv7.4^W242X^-expressing cells from the bottom of a culture dish within 24 h after the application of doxycycline. Similar rounding was also often seen in Kv7.4^A349fs^-expressing cells within 40 h after the application of doxycycline (examples shown in Fig. 1E). We also had difficulty with whole-cell patch-clamp recording in these cells, indicating impaired cell membrane integrity. Unhealthiness of Kv7.4^W242X^-expressing cells was also reflected in their significantly depolarized V_res_ (Fig. 1D). Co-expression of Kv7.4^W242X^ and Kv7.4^WT^ also resulted in cell rounding/detachment and made whole-cell recording challenging. Expression of Kv7.4^Q71fs^ and Kv7.4^A349fs^ did not affect the function of co-expressed Kv7.4^WT^ (Fig. 1F). The significantly depolarized V_res_ found in cells co-expressing Kv7.4^W242X^ and Kv7.4^WT^ (‘WT/W242X’ in Fig. 1F) may be seen as supporting evidence for dominant-negative inhibition. However, it is possible that this apparent dominant-negative inhibitory effect may be ascribed to impaired membrane integrity in unhealthy cells (see below). It should also be pointed out that some cells show low V_res_ that is comparable to WT/WT. As this WT/WT-like V_res_ may be due to a greater expression of Kv7.4^WT^ compared with Kv7.4^W242X^ in those particular cells, we repeated the whole-cell recordings using additionally generated cell lines in which Kv7.4^WT^ and the truncated Kv7.4 variants are co-expressed at similar levels using the self-cleaving P2A linkage (‘WT-P2A-Q71fs’, ‘WT-P2A-W242X’ and ‘WT-P2A-A349fs’ in Fig. 1F). However, WT/WT-like V_res_ was still observed in some cells co-expressing Kv7.4^WT^ and Kv7.4^W242X^ (Fig. 1F), casting doubt over the ability of Kv7.4^W242X^ to exert a dominant-negative inhibitory effect on Kv7.4^WT^.

### Kv7.4^Q71fs^, Kv7.4^W242X^ and Kv7.4^A349fs^ do not form a heteromultimer with Kv7.4^WT^

**Fig. 2.**
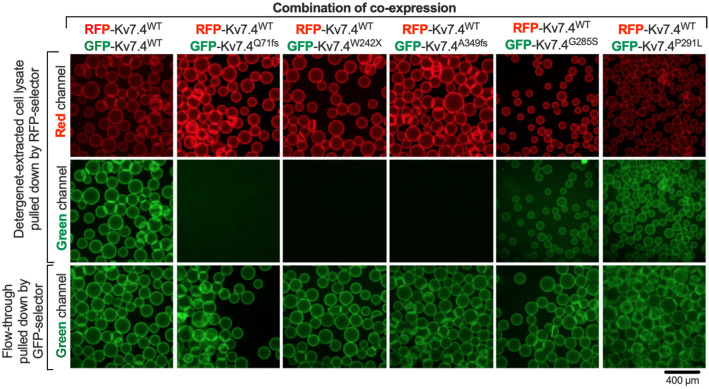
**Co-immunoprecipitation assay to assess the binding ability of the truncated Kv7.4 variants to wild-type Kv7.4.** RFP-tagged Kv7.4^WT^ was co-expressed with each of the GFP-tagged truncated Kv7.4 variants in HEK293T cells, solubilized in a mild detergent-containing buffer and pulled down with anti-RFP-conjugated beads (RFP-selector). Red fluorescence-positive beads (top panels) indicate successful pull down of RFP- Kv7.4^WT^. Detection of green fluorescence indicates binding between WT and a Kv7.4 variant (not seen for all the three truncated Kv7.4 variants). The unbound fractions (flow-through) were incubated and pulled down with GFP-selector. Green fluorescence-positive beads indicate the presence of co-expressed GFP-tagged Kv7.4 constructs that did not bind to RFP- Kv7.4^WT^ (bottom panels). All fluorescence images were taken using the same image acquisition setting.

In order to confirm the inability of Kv7.4^Q71fs^, Kv7.4^W242X^ and Kv7.4^A349fs^ to form a complex with Kv7.4^WT^ to exert a dominant-negative inhibitory effect, we established cell lines co-expressing RFP-tagged Kv7.4^WT^ and GFP-tagged Kv7.4^WT^ (Leitner et al., 2012), or each of the three GFP-tagged truncated Kv7.4 variants (Table S1). The cells were lysed, and detergent-solubilized RFP-tagged Kv7.4^WT^ was captured using anti-RFP-conjugated beads. As expected, GFP-tagged Kv7.4^WT^ co-precipitated with RFP-tagged Kv7.4^WT^ (Fig. 2). However, none of the three truncated Kv7.4 variants co-precipitated with RFP-tagged Kv7.4^WT^ (Fig. 2), confirming that these truncated Kv7.4 variants do not complex with Kv7.4^WT^ to exert dominant-negative inhibitory effects. On the other hand, our co-precipitation assay detected the binding of two selected Kv7.4 missense variants, Kv7.4^G285S^ and Kv7.4^P291L^, to Kv7.4^WT^ (Fig. 2). The V_res_ values of HEK293T cells singly expressing Kv7.4^G285S^ or Kv7.4^P291L^ were −21±17 mV (*n*=6) and −14±9 mV (*n*=5) (mean±s.d.), respectively, which were statistically indistinguishable (*P*>0.05) from that of untransfected HEK293T cells (−20±8 mV, *n*=10). These observations do not oppose the dominant-negative inhibition-based pathological mechanism that has been experimentally demonstrated for many missense Kv7.4 variants (Baek et al., 2011; Gao et al., 2013; Jung et al., 2019; Kharkovets et al., 2006; Kim et al., 2011; Kubisch et al., 1999; Mencía et al., 2008; Shin et al., 2019).

### Kv7.4^Q71fs^, Kv7.4^W242X^ and Kv7.4^A349fs^ are cytotoxic and induce cell death

**Fig. 3.**
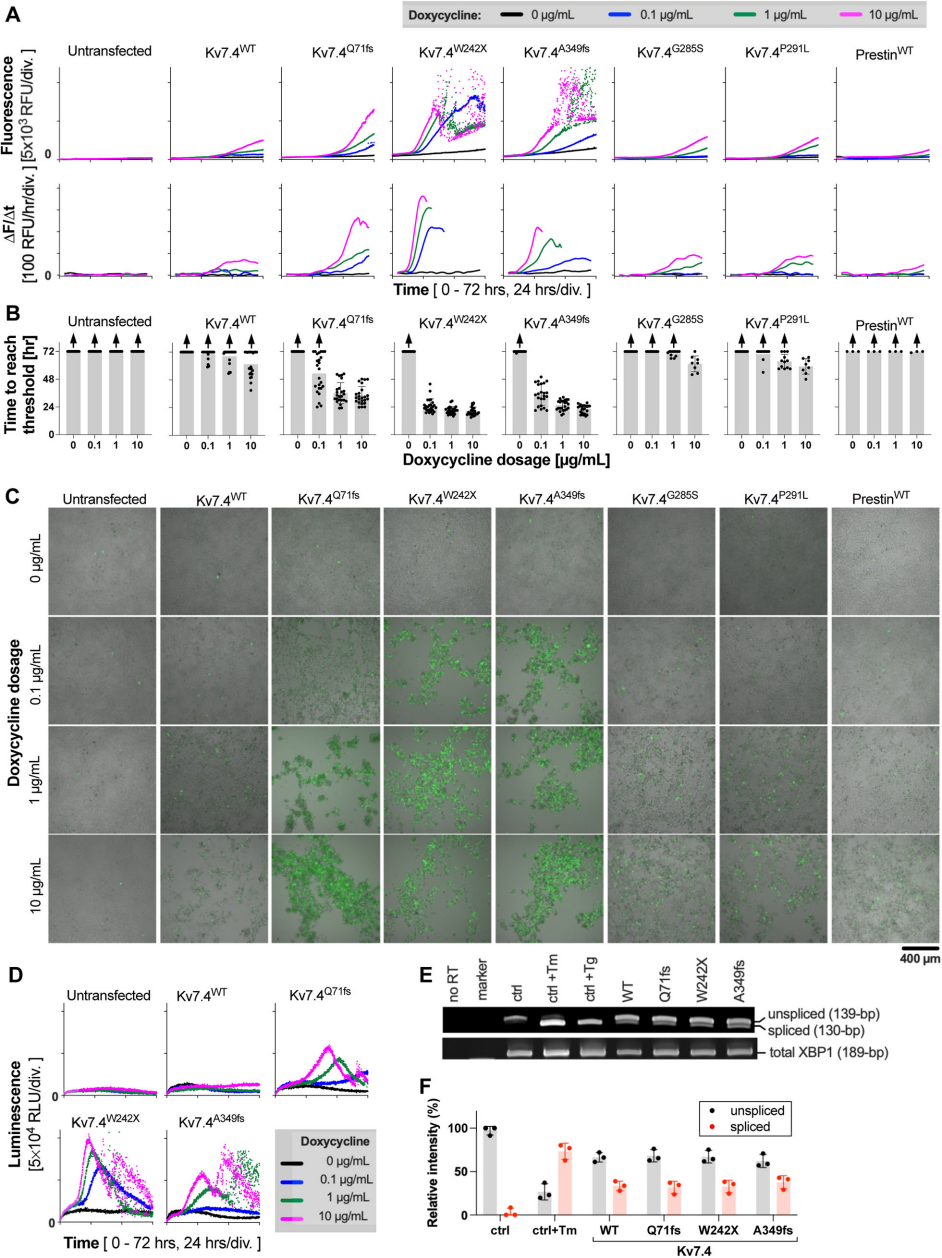
**Plate reader-based real-time cell death assays to assess cytotoxicity of the Kv7.4 variants.** (A) CellTox Green Cytotoxicity assay. The scattered readings often seen at later time points in Kv7.4^W242X^ and Kv7.4^A349fs^ are probably due to dying or dead cells detached from the bottoms of the wells. Expression of Kv7.4 constructs were induced in a 96-well plate by the addition of doxycycline (0-10 µg/ml) at the beginning of the assays (at time zero), and cell death-indicating fluorescence was monitored for 72 h (37°C, 5% CO_2_). RFU, relative fluorescence unit. ΔF/Δt (lower panels) is the rate of change in the CellTox Green fluorescence (F). (B) Summaries of the CellTox Green Cytotoxicity assay. The onset of detecting cell death (threshold) is defined as the initial background fluorescence (<5 h) plus 1000 RFU. The maximum value, 72 h, was assigned to those that did not reach the threshold by the end of the 72-h long assay. Upward arrows indicate underestimation of the mean values due to inclusion of such ‘maxed out’ data. Data are mean±s.d. (C) Cell images taken after the CellTox Green Cytotoxicity assay at 72 h. Bright-field and fluorescence images are merged in each panel. All fluorescence images were taken using the same image acquisition setting. Fig. S1 shows the same images with a higher resolution. (D) RealTime-Glo Annexin V Apoptosis assay. Expression of various Kv7.4 constructs was induced in a 96-well plate by the addition of doxycycline (0-10 µg/ml) at the beginning of the assays (at time zero), and luminescence was monitored for 72 h (37°C, 5% CO_2_). RLU, relative luminescence unit. Results from one out of six sets of experiments are shown. (E) Detection of ER stress-induced splicing of XBP1 mRNA. Total RNAs were isolated, reverse transcribed, and portions of spliced (130 bp) and unspliced (139 bp) XBP1 cDNAs amplified by PCR. A portion not including the ER stress-dependent splice site was also amplified (total XBP1, 189 bp). The PCR products were analyzed by acrylamide gel electrophoresis. ‘no RT’ is negative PCR control without reverse transcription (RT). ‘ctrl’ indicates untransfected HEK293T cells (1 µg/ml doxycycline for 6 h). ‘Tm’ and ‘Tg’ indicate tunicamycin (2 µg/ml for 6 h) and thapsigargin (300 nM for 6 h), respectively, which were used as positive controls of ER stress induction. Expression of the Kv7.4 constructs was induced by 1 µg/ml doxycycline for 6 h. (F) A summary of the ratios of spliced and unspliced bands. The relative band intensities were determined by double Gaussian fitting.

The aforementioned difficulty with whole-cell recordings in Kv7.4^W242X^-expressing cells and their significantly depolarized V_res_ compared to untransfected cells (Fig. 1D) imply that the expression of Kv7.4^W242X^ impairs cell viability. Although we did not notice significant difficulty with whole-cell recordings in Kv7.4^Q71fs^- and Kv7.4^A349fs^-expressing cells (∼24 h after application of doxycycline), their mean V_res_ values were depolarized compared to untransfected cells (Fig. 1D), suggesting that these two truncated Kv7.4 variants may also be cytotoxic and impair cell viability. To explore this possibility, we performed a CellTox Green Cytotoxicity assay. In this assay, the cell membrane impermeable non-fluorescent dye goes into dying cells with impaired cell membrane integrity and becomes fluorescent upon binding to DNA. The expression of Kv7.4 constructs was induced by various dosages of doxycycline, and the cell death-indicating green fluorescence (CellTox Green) was monitored over time (Fig. 3A, upper panels). Compared to the untransfected cell control, the cell-death-indicating green fluorescence was detected even in Kv7.4^WT^-expressing cells. However, cells expressing the truncated Kv7.4 variants showed much greater CellTox Green fluorescence. The earliest onset of the large fluorescence change (<24 h after doxycycline application) found in Kv7.4^W242X^-expressing cells accounted for the difficulty we experienced in whole-cell recording in these cells. Plotting the time derivative of the CellTox Green fluorescence, ΔF/Δt, against time accentuated the difference in cytotoxicity between WT and variants (Fig. 3A, lower panels). To quantitatively compare the cytotoxicity among Kv7.4 constructs, we defined the threshold of detecting cell death as the basal fluorescence plus 1000 relative fluorescence units (RFU) (Fig. 3B). The observed dependence of CellTox Green fluorescence on the doxycycline dosage (Fig. 3A,B) further ascertains that the expression of the truncated Kv7.4 variants is indeed the cause of cell death. Cytotoxic effects of the truncated Kv7.4 variants also manifested in cell images taken after the CellTox Green Cytotoxicity assay. Unlike untransfected cells that reached over-confluency by the end of the 72-h assay, cells expressing the truncated Kv7.4 variants were typically rounded up, clumped together, detached from the bottom of the wells and, thus, never reached confluency (Fig. 3C) (the same images with a higher resolution are provided in Fig. S1). Time-dependent changes in CellTox Green fluorescence in cells expressing Kv7.4^G285S^ and Kv7.4^P291L^ were similar to those seen in Kv7.4^WT^-expressing cells (Fig. 3A,B). We also performed the CellTox Green Cytotoxicity assay for cells expressing prestin (SLC26A5), which is another membrane protein abundantly expressed in OHCs. Some CellTox Green fluorescence was detected in prestin-expressing cells at the highest doxycycline dosage, but it was much smaller compared to Kv7.4^WT^-expressing cells (Fig. 3A,B, far right panels).

The cytotoxicity of the three truncated Kv7.4 variants was further examined using a RealTime-Glo Annexin V Apoptosis Assay, which uses annexin V to detect phosphatidylserine on the outer leaflet of the cell membrane. An increase of phosphatidylserine on the outer leaflet of the cell membrane is indicative of apoptotic cells (Martin et al., 1995). As shown in Fig. 3D, this assay also found doxycycline dose-dependent cytotoxicity in cells expressing Kv7.4^Q71fs^, Kv7.4^W242X^ and Kv7.4^A349fs^, affirming that these truncated Kv7.4 variants are indeed cytotoxic.

### Expression of the Kv7.4 constructs triggers endoplasmic reticulum stress

As Kv7 channels are membrane proteins that mature in the endoplasmic reticulum (ER), it is conceivable that the observed intense cell death induced by some Kv7 variants may be ascribed to accumulations of misfolded Kv7 proteins in the ER, resulting in chronic irreversible ER stress. In fact, a previous study provided experimental evidence supporting ER retention for several Kv7.4 variants (Kim et al., 2011). In order to capture a sign of ER stress free from likely complications associated with cell death (e.g. degradation or loss of stress-indicating signals upon cell death), we collected cells 6 h after the application of doxycycline (1 µg/ml) and extracted RNA for examining ER stress-dependent splicing of XBP1 mRNA (Yoshida et al., 2001) (Fig. 3E,F). Spliced XBP1 mRNA was detected in cells treated with the well-known ER stress-inducing reagents tunicamycin and thapsigargin (positive controls) but was minimal in untransfected cells (negative control), as expected. Cells expressing Kv7.4^WT^, Kv7.4^Q71fs^, Kv7.4^W242X^ or Kv7.4^A349fs^ showed clearly detectable spliced XBP1 bands, suggesting that expression of the Kv7.4 constructs do indeed induce ER stress.

### Some truncated Kv7.1 variants lacking the C-terminal tetramerization region also induce cell death

**Fig. 4.**
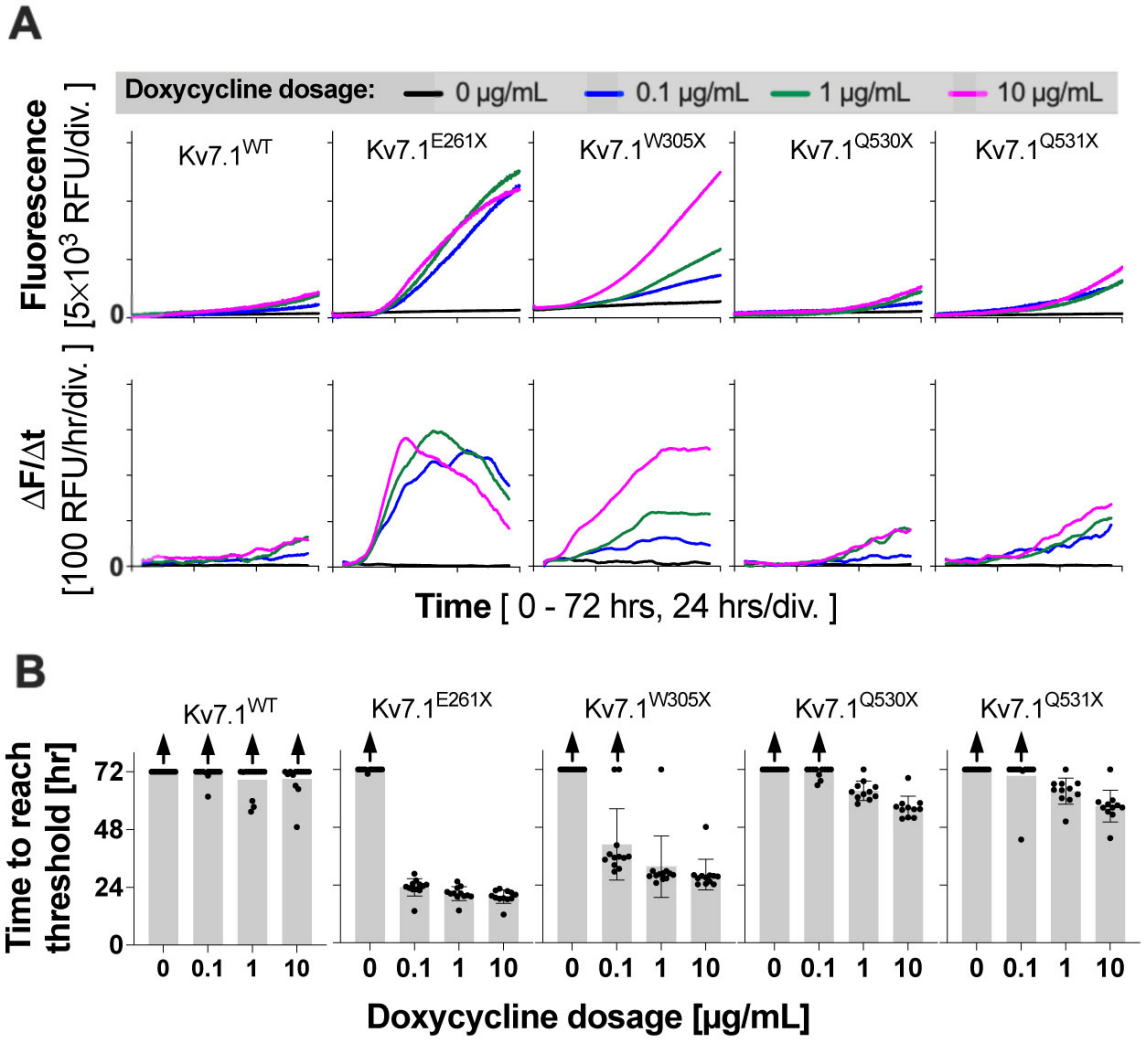
**CellTox Green Cytotoxicity assay for Kv7.1 variants.** (A) Expression of Kv7.1 constructs were induced in a 96-well plate by the addition of doxycycline (0-10 µg/ml) at the beginning of the assays (at time zero), and cell death-indicating fluorescence was monitored for 72 h (37°C, 5% CO_2_). RFU, relative fluorescence unit. ΔF/Δt (lower panels) is the rate of change in the CellTox Green fluorescence (F). (B) Summaries of the CellTox Green Cytotoxicity assay. The onset of detecting cell death (threshold) is defined as the initial background fluorescence (<5 h) plus 1000 RFU. The maximum value, 72 h, was assigned to those that did not reach the threshold by the end of the 72-h long assay. Upward arrows indicate underestimation of the mean values due to inclusion of data that did not reach the threshold by the end of the 72-h long assay. Data are mean±s.d.

*KCNQ1* encodes the homotetrameric voltage-dependent potassium ion channel Kv7.1, which co-assembles with its regulatory KCNE subunits. The molecular architecture of Kv7.1 is quite similar to that of Kv7.4 (Fig. 1A). The vast majority of Kv7.1 variants are associated with dominantly inherited long QT syndrome (LQTS), but some are inherited recessively and also associated with hearing loss in addition to LQTS (Jervell and Lange-Nielsen syndrome, JLNS) (Neyroud et al., 1997). The fact that patients with heterozygous JLNS-associated *KCNQ1* variants suffer from neither LQTS nor hearing loss suggests that one functional *KCNQ1* allele is sufficient for maintaining normal cardiac and auditory function. We suspect that many dominantly inherited truncated Kv7.1 variants are also cytotoxic. In order to explore this possibility, we repeated the CellTox Green Cytotoxicity assay for four selected truncated Kv7.1 variants, Kv7.1^E261X^, Kv7.1W^305X^, Kv7.1^Q530X^ and Kv7.1^Q531X^ (Stenson et al., 2017). All these Kv7.1 variants lack the C-terminal tetramerization region (Fig. 1A). It was experimentally demonstrated that Kv7.1^Q530X^ does not exert a dominant-negative inhibitory effect on Kv7.1^WT^ in cells co-expressing Kv7.1^Q530X^ and Kv7.1^WT^ at similar levels (Huang et al., 2001; Westenskow et al., 2004). Consistently, it was demonstrated that Kv7.1^Q530X^ does not bind to Kv7.1^WT^ (Wilson et al., 2005). It is probable that the other three Kv7.1 variants, which are truncated similarly (Kv7.1^Q531X^) or shorter (Kv7.1^E261X^ and Kv7.1W^305X^) compared to Kv7.1^Q530X^, are also incapable of exerting a dominant-negative inhibitory effect on Kv7.1^WT^. As shown in Fig. 4, these truncated Kv7.1 variants showed doxycycline dose-dependent cytotoxicity to varying degrees. Of note, the lowest cytotoxicity was found in Kv7.1^Q530X^, which is associated with recessively inherited JLNS (Tranebjaerg et al., 1999).

### Pharmacological mitigation of the cytotoxic effect of the truncated Kv7.4 variants

**Fig. 5.**
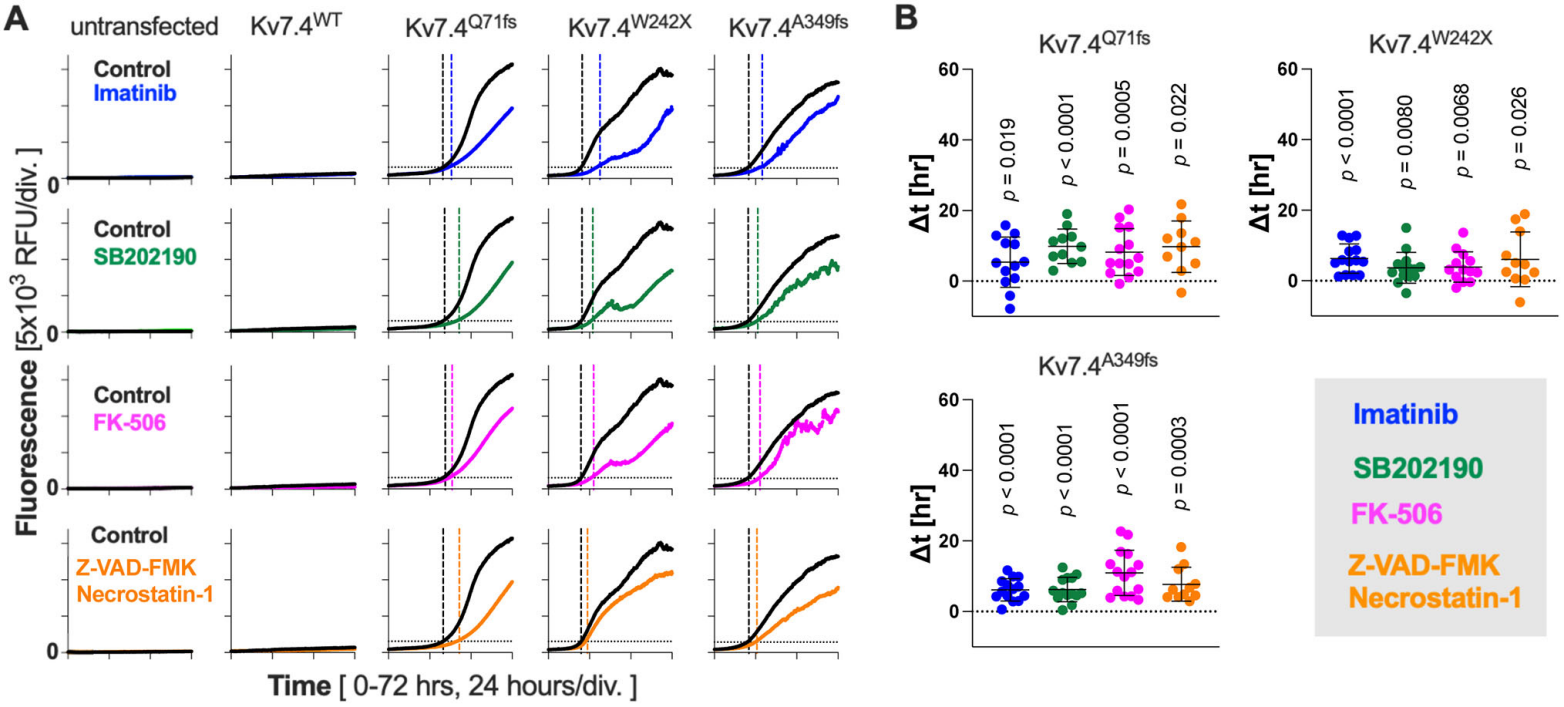
**Ameliorative effects of autophagy inducers against the cytotoxic Kv7.4 variants.** (A) A CellTox Green Cytotoxicity assay was performed in the presence of autophagy inducers (imatinib, SB202190 or FK-506) or an apoptosis/necrosis inhibitor cocktail (Z-VAD-FMK and necrostatin-1) added at time zero. Doxycycline (1 µg/ml) was also added at time zero to induce expressions of Kv7.4 constructs. Horizontal broken lines indicate threshold fluorescence that was defined as the mean fluorescence within the first 5 h plus 1000 RFU. Vertical broken lines indicate time points at which CellTox Green fluorescence traces and the horizontal broken lines are crossed (thresholds). The difference between a colored broken line and a vertical black broken line, Δt (colored minus black), was determined for each assay. (B) Summaries of Δt. The horizontal solid lines indicate the mean Δt values±s.d. Statistical significance was determined by one-sample *t*-tests with respect to Δt=0 (indicated by broken horizontal lines).

**Fig. 6.**
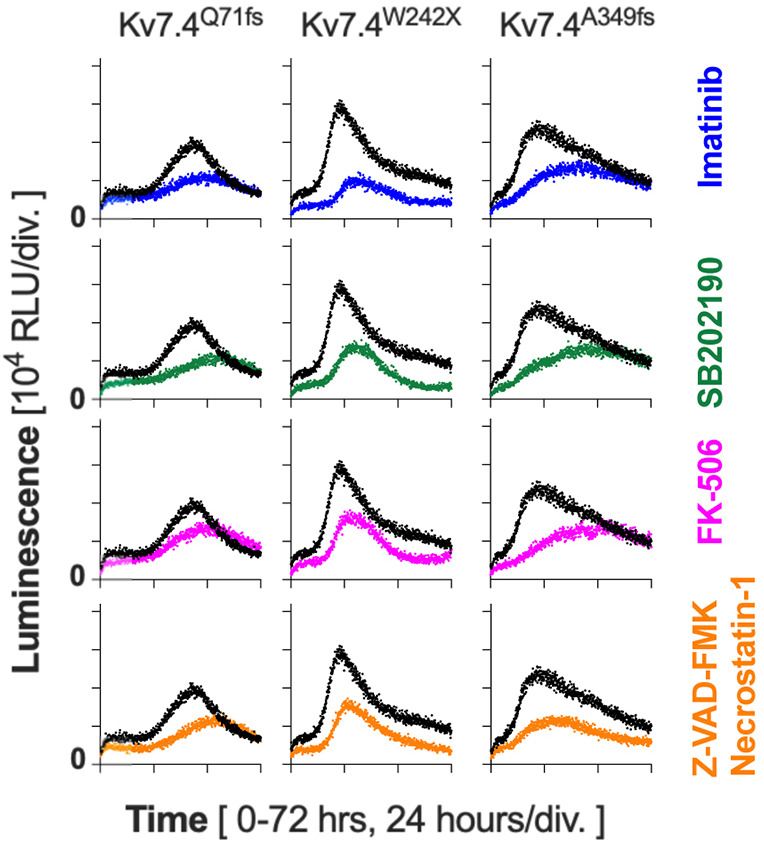
**Confirmation of the ameliorative effects of the autophagy inducers by the RealTime-Glo Annexin V Apoptosis assay.** Effects of autophagy inducers (imatinib, SB202190 and FK-506) and an apoptosis/necrosis inhibitor cocktail (Z-VAD-FMK and necrostatin-1) were assessed using a RealTime-Glo Annexin V Apoptosis assay. Expression of the Kv7.4 variants was induced by the addition of doxycycline (1 μg/ml) at time zero. Drugs were also added at time zero, except for controls (black traces). Luminescence was monitored for 72 h (37°C, 5% CO_2_). RLU, relative luminescence unit. Representative results from one out of four sets of experiments are shown.

Doxycycline dosage dependence of the cytotoxic effects (Figs 3, 4) indicates that expression and accumulation of the truncated Kv7 proteins are the cause of cellular stress and subsequent cell death. If true, augmentation of a cellular pathway(s) responsible for clearing the accumulated proteins would ameliorate the cytotoxic effect of the Kv7 variants. To explore this possibility, we performed a CellTox Green Cytotoxicity assay in the presence of a variety of autophagy-modulating drugs. We found that applications of three autophagy inducers, imatinib mesylate (Ertmer et al., 2007), SB202190 (Schwartz et al., 2018) and FK-506 (Kim et al., 2017), notably delayed the onset of CellTox Green fluorescence and suppressed its rate of increase in cells expressing the truncated Kv7.4 variants (Fig. 5A). An apoptosis/necrosis inhibitor cocktail containing Z-VAD-FMK and necrostatin-1 also showed an ameliorative effect, further affirming the cell death-inducing cytotoxic effect of the truncated Kv7.4 variants. We determined the onset times of cell death (indicated by vertical broken lines in Fig. 5A) by setting arbitrary thresholds (the basal fluorescence plus 1000 RFU, indicated by horizontal broken lines in Fig. 5A). The effects of the drugs were then quantified by determining the delays of the onset times by drug applications (Δt). As shown in Fig. 5B, one-sample *t*-tests found that the ameliorative effects of the three autophagy inducers and the apoptosis/necrosis inhibitor cocktail were statistically significant. We also performed the annexin V-based apoptosis assay and qualitatively confirmed the ameliorative effects of the autophagy inducers (Fig. 6).

### Inhibition of basal autophagy aggravates cytotoxicity

**Fig. 7.**
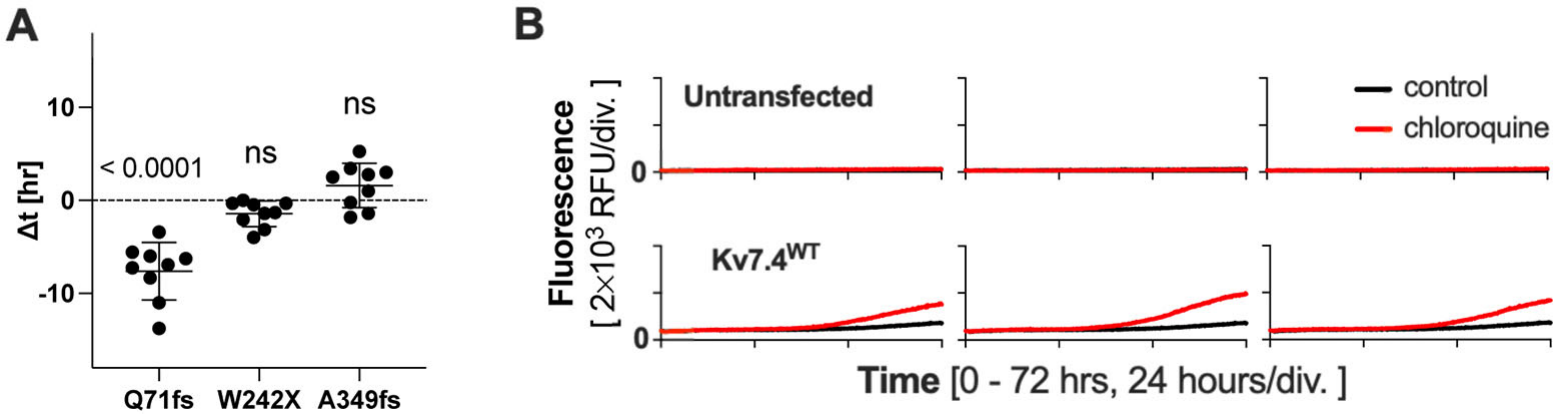
**The autophagy inhibitor chloroquine aggravates the cell death-inducing cytotoxicity of Kv7.4 and its truncated variant Kv7.4^Q71fs^.** Doxycycline (1 µg/ml) and chloroquine diphosphate (10 µM) were added at time zero, and cell death-indicating fluorescence (CellTox Green) was monitored for 72 h (37°C, 5% CO_2_). (A) The effect of chloroquine on cells expressing Kv7.4^Q71fs^, Kv7.4^W242X^ or Kv7.4^A349fs^ was quantified. Statistical significance was determined by one-sample *t*-tests with respect to Δt=0 (indicated by broken horizontal lines). ns, not significant. (B) The effect of chloroquine on untransfected (upper panels) and Kv7.4^WT^-expressing HEK293T cells (lower panels). Three examples are shown for each. The total numbers of replicates were ten and nine for untransfected control and Kv7.4^WT^, respectively. RFU, relative fluorescence unit.

A forced large expression of any protein could impose significant cellular stress and impair cell viability. In fact, we found that the expression of even Kv7.4^WT^ could induce ER stress and induce a small but non-negligible degree of cell death (Fig. 3). We explored the physiological relevance of this finding, given the high abundance of the Kv7.4 protein in OHCs. It is conceivable that the cytotoxicity of Kv7.4^WT^ is largely counteracted by endogenous autophagy activity. If true, inhibition of endogenous autophagy would render cells vulnerable to cytotoxicity associated with a large expression of Kv7.4^WT^. It is also conceivable that inhibition of autophagy may aggravate the cytotoxic effects of the truncated Kv7.4 variants. To test this, we repeated the CellTox Green Cytotoxicity assay in the presence of an autophagy inhibitor, chloroquine (Klionsky et al., 2016; Mauthe et al., 2018; Redmann et al., 2017) (Fig. 7). The effect of chloroquine was quantified (Fig. 7A) as in Fig. 5, except for Kv7.4^WT^ and the untransfected control, which often did not reach the cell death detection threshold within 72 h. The results of Kv7.4^WT^ and the untransfected control are thus presented qualitatively by showing three examples for each (Fig. 7B). As shown, application of chloroquine noticeably aggravated the cytotoxic effect of Kv7.4^WT^, affirming the involvement of autophagy and its protective role against a large expression of the Kv7.4 protein. This observation is in line with a recent study showing that chloroquine specifically damages OHCs in mice (Davis et al., 2020). The aggravating effect of chloroquine was also found in Kv7.4^Q71fs^ (Fig. 7A), further affirming the presence of the basal autophagy and its protective role. On the contrary, the application of chloroquine did not exacerbate the progression of cell death induced by Kv7.4^W242X^ and Kv7.4^A349fs^ (Fig. 7A). These observations likely reflect the severe cytotoxicity of Kv7.4^W242X^ and Kv7.4^A349fs^ (Fig. 3), which would quickly consume the basal capacity of autophagy flux.

### The expression of truncated Kv7.4 variants also induces cell death in HEI-OC1 and LLC-PK1 cells

**Fig. 8.**
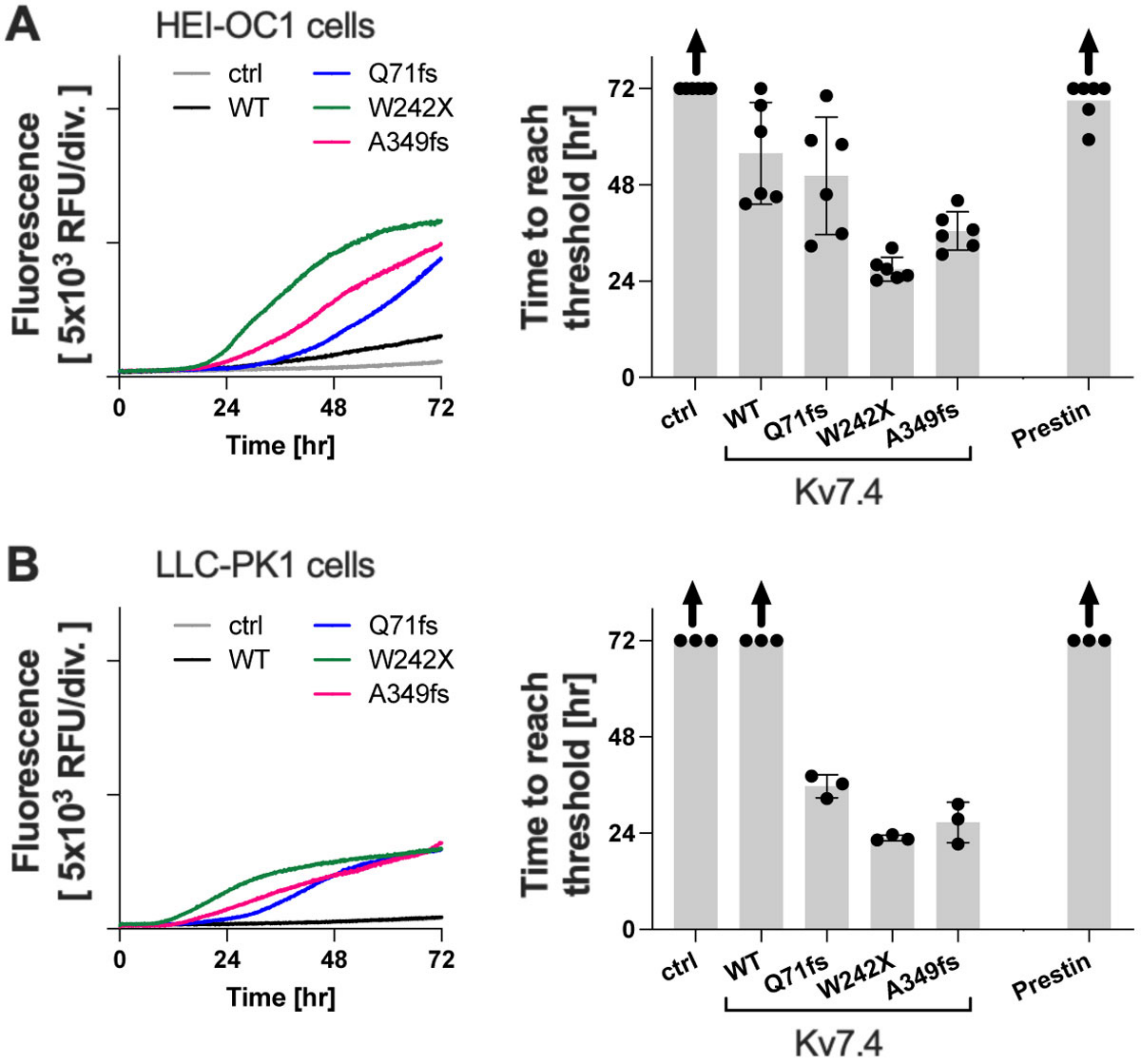
**CellTox Green Cytotoxicity assay in HEI-OC1 and LLC-PK1 cells.** (A,B) The pathophysiological relevance and generality of the cell death-inducing cytotoxicity of the truncated Kv7.4 variants were examined in HEI-OC1 cells (A) and LLC-PK1 epithelial cells (B). The left panels show examples of CellTox Green Cytotoxicity assays. Expression of Kv7.4 or prestin constructs was induced in a 96-well plate by the addition of doxycycline (1 µg/ml) at the beginning of the assays (at time zero), and cell death-indicating fluorescence was monitored for 72 h (37°C, 5% CO_2_). Right panels show summaries of the CellTox Green Cytotoxicity assay. The onset of detecting cell death (threshold) is defined as the initial background fluorescence (<5 h) plus 1000 RFU. The maximum value, 72 h, was assigned to those that did not reach the threshold by the end of the 72-h long assay. Upward arrows indicate underestimation of the mean values due to inclusion of maxed out (72-h maximum) data that did not reach the threshold by the end of the assay. RFU, relative fluorescence unit. Data are mean±s.d.

As Kv7.4 is most abundantly expressed in OHCs in the inner ear, and OHC dysfunction and subsequent loss are believed to be the primary cause of DFNA2 hearing loss, we repeated the CellTox Green Cytotoxicity assay for Kv7.4^WT^, Kv7.4^Q71fs^, Kv7.4^W242X^, Kv7.4^A349fs^ and prestin^WT^ in an HEI-OC1 cell line that was derived from murine inner ear (Kalinec et al., 2003). We also repeated the experiment in an LLC-PK1 cell line that was derived from porcine kidney (Hull et al., 1976) to test the generality of our finding. As shown in Fig. 8A,B, the results obtained in these two cell lines look qualitatively very similar to those obtained in HEK293T cells (Fig. 3). These observations warrant future studies to further investigate the cytotoxicity of the truncated Kv7.4 variants in animal models.

## DISCUSSION

Both Kv7.1 and Kv7.4 form homotetramers, and this formation is mediated by the C-terminal helices C and D (HC and HD, Fig. 1A). Kv7 variants lacking this C-terminal tetramerization region probably do not form multimers with the Kv7^WT^ subunit. In this study, we experimentally confirmed that the three truncated Kv7.4 variants lacking the C-terminal region are indeed incapable of forming a heteromultimer with the Kv7.4^WT^ subunit (Fig. 2). Lack of multimerization ability to exert a dominant-negative inhibitory effect was also demonstrated in previous studies for a JLNS-associated truncated Kv7.1 variant, Kv7.1^Q530X^ (Huang et al., 2001; Westenskow et al., 2004; Wilson et al., 2005). The presence of recessively inherited truncated Kv7.1 and Kv7.4 variants, such as Kv7.1^Q530X^ and Kv7.4^A349fs^, suggests that one functional *KCNQ1/KCNQ4* allele is sufficient for maintaining normal cardiac and/or auditory functions, thus questioning a haploinsufficiency-based pathological mechanism. A cytotoxicity-based pathological mechanism, on the other hand, provides a straightforward explanation for the observed graded severity of disease phenotypes associated with various truncated Kv7 variants and their modes of inheritance. It has long been realized that the severity of cardiac phenotypes is not always correlated with the corrected QT interval (QTc), making diagnosis solely based on QTc values problematic (Chen et al., 2003; Priori et al., 1999; Vincent, 2000; Vincent et al., 1992). For example, a normal-to-borderline resting QTc interval of 0.46±0.2 s (*n*=8) was found in patients with Kv7.1^W305X^ (c.914G>A) (Chen et al., 2003), which seems reasonable given the probable inability of this truncated variant to exert a dominant-negative inhibitory effect and the lack of need for having two functional KCNQ1 alleles for maintaining normal cardiac function (see above). However, the proband with this Kv7.1 variant died suddenly at the age of 16, and two other affected members experienced syncopal episodes (Chen et al., 2003). It is conceivable that the large cytotoxicity found in Kv7.1^W305X^ (Fig. 4) may underlie the severe cardiac phenotypes found in these patients. The smallest cytotoxicity, of the four truncated Kv7.1 variants tested, was found in the recessively inherited Kv7.1^Q530X^ (Fig. 4), and is compatible with such a view. However, the large cytotoxicity found in Kv7.4^A349fs^ (Fig. 3) contradicts the fact that this variant is inherited recessively. Our experimental efforts using a heterologous expression system has limitations in terms of predicting the expression of Kv7.4 variant proteins *in vivo*. Although animal models need to be generated in future studies to fully define the pathological roles of Kv7.4 variants, it would be reasonable to speculate at this point that the pattern of inheritance (from fully recessive to fully dominant) of many, if not most, truncated Kv7 variants may be determined largely by the severity of their anticipated cytotoxicity.

Several preceding studies reported reduced membrane targeting or intracellular retention of Kv7.4 variants (Mencía et al., 2008; Kim et al., 2011; Heidenreich et al., 2012; Spitzmaul et al., 2013). Kim et al. (2011) showed colocalization of Kv7.4 variants and an ER marker, P450, which is indicative of ER retention and thus may imply ER stress. However, these previous reports have not led to subsequent experimental efforts to explore cytotoxicity in Kv7.4 variants, as the direct consequence of intracellular retention is a reduction of the overall potassium ion channel activity, and because it is widely accepted that OHC degeneration in DFNA2 is ascribed to chronic depolarization of the cells due to reduced or absent Kv7.4-mediated potassium ion conductance (Kharkovets et al., 2006). To the best of our knowledge, cell death-inducing cytotoxicity has never been demonstrated for any Kv7.4 variants. We fortuitously began to explore cell death-inducing cytotoxicity for Kv7.4 variants because of the difficulty we faced at the earliest stage of this study in establishing stable cell lines that could constitutively express truncated Kv7.4 variants for functional assays, especially for Kv7.4^W242X^. The use of the doxycycline-inducible expression system was crucial to unequivocally identify and describe the cell death-inducing severe cytotoxicity reported herein.

We detected splicing of XBP1 mRNA, which is one of the hallmarks of ER stress, in cells expressing Kv7.4^WT^, Kv7.4^Q71fs^, Kv7.4^W242X^ and Kv7.4^A349fs^ (Fig. 3E,F), suggesting that the induction of ER stress response preceded the observed cell death. It seems evident that the severity of cell death-inducing cytotoxicity depends on the amounts of Kv7.4 proteins produced (Fig. 3B), but it largely differs among the Kv7.4 constructs under the same doxycycline dosage conditions. It is conceivable that this difference may be ascribed to the distinct stability (e.g. half-life of stress-inducing aggregates) of the variant proteins, which affects the clearance rate. The fact that autophagy modulators can ameliorate or exacerbate the cytotoxic effects of the Kv7.4 variants (Figs 5-7) does not oppose such a scenario.

Two missense Kv7.4 variants, Kv7.4^G285S^ and Kv7.4^P291L^, have an intact C-terminal cytosolic domain. We confirmed that these missense variants can interact with Kv7.4^WT^ (Fig. 2). Gly^285^ and Pro^291^ are located in the pore-forming α-helix (PH in Fig. 1A) and are conserved among the Kv7 family members. The importance of these residues for potassium ion channel function is structurally foreseeable and strongly suggested by the presence of multiple disease-associated missense variants changing these residues in addition to Kv7.4^G285S^ and Kv7.4^P291L^ [i.e. G314A, G314R, G314D, G314C, G314S, P320A, P320H and P320S for Kv7.1; G279C, G279S, P285H, P285S and P285T for Kv7.2; and G285C and P291S for Kv7.4] (Stenson et al., 2017). A dominant-negative inhibitory effect induced by missense changes of these conserved glycine and proline residues has been experimentally demonstrated previously (Baek et al., 2011; Kharkovets et al., 2006; Kim et al., 2011; Kubisch et al., 1999; Li et al., 2009; Thomas et al., 2010; Westenskow et al., 2004). Compared to severe dominant-negative inhibition induced by missense changes of the glycine residue, missense changes of the proline residue result in relatively mild dominant-negative inhibition, which may account for the semi-dominant inheritance of Kv7.4^P291L^ (Ramzan et al., 2019). In any case, our study does not challenge the dominant-negative inhibition-based pathological mechanism for Kv7.4^G285S^ and Kv7.4^P291L^, because the cytotoxicity of these missense variants was similar to that of Kv7.4^WT^ (Fig. 3). However, one should not deduce from these limited observations that the large cytotoxicity reported herein should only be suspected for truncated Kv7 variants. Currently, more than 1000 disease-associated genetic variants are identified in *KCNQ1-5* (Stenson et al., 2017). It would be worth screening for the cytotoxicity of any dominantly inherited *KCNQ* variants that are presumed to affect the amino acid sequences of the Kv7 proteins. In fact, a previous study reported a cytotoxic effect of Kv7.2^M518V^ (Kim et al., 2018). It is crucial to identify and distinguish cytotoxic variants from the others because the currently pursued pharmacological strategy to augment reduced residual Kv7 channel activity (Barrese et al., 2018; Imbrici et al., 2016; Leitner et al., 2012; Miceli et al., 2018; Salata et al., 1998; Shin et al., 2019; Tian et al., 2014; Wuttke and Lerche, 2006; Xiong et al., 2007; Xu et al., 2002) would not likely benefit patients with cytotoxic Kv7 variants.

This study identified several autophagy-enhancing reagents that mitigate the cytotoxic effect of the truncated Kv7.4 variants (Figs 5,6). Given the high abundance of the Kv7.4 protein in OHCs and its small but non-negligible cytotoxicity (Fig. 3), it is conceivable that the basal endogenous autophagy activity in OHCs may be kept at a relatively high level to counteract the cytotoxicity associated with the high expression of the Kv7.4 protein. Consistent with such views, the autophagy inhibitor chloroquine is reported to induce hair cell loss (Davis et al., 2020). Hair cell loss was also found in conditional autophagy-deficient mice (Fujimoto et al., 2017). A pharmacological intervention to maintain or augment the endogenous autophagy activity may delay or prevent age-related hearing loss (presbycusis).

The *in vitro* cytotoxicity assays used in this study are useful for rapidly identifying promising anti-cytotoxic drugs but they have limitations in terms of examining potential adverse effects of drugs on hearing. For example, the reported ototoxicity of imatinib (Attili et al., 2008; Wasif et al., 2016) cannot be evaluated in the *in vitro* cytotoxicity assays. Generation of animal models expressing cytotoxic Kv7.4 variants will be crucial in future studies to fully characterize drugs that show potential in the *in vitro* cytotoxicity assays.

The main focus of the present study was on the three truncated Kv7.4 variants, and we unexpectedly found cell death-inducing cytotoxicity in these variants, as well as in some Kv7.1 variants. Given the structural similarity shared among all the Kv7 family members, it is likely that there are many other cytotoxic Kv7 variants outside of Kv7.1 and Kv7.4, and that a pharmacological intervention proven effective for a certain Kv7 variant will also work effectively for other cytotoxic Kv7 variants. Our finding thus has the potential to develop a generalizable pharmacological strategy for many dominantly inherited pathogenic Kv7 variants.

## MATERIALS AND METHODS

### Generation of stable cell lines that express various Kv7 protein constructs

Plasmids containing cDNAs that encode N-terminally GFP-tagged human Kv7.4 and N-terminally RFP-tagged human Kv7.4 were generous gifts from Dr Michael Leitner (Philipps University, Marburg, Germany), which were used in their previous study (Leitner et al., 2012). A plasmid containing cDNA that encodes human Kv7.1 was purchased from Addgene (plasmid 111452). Various Kv7.4 and Kv7.1 constructs were generated, based on these cDNAs, by PCR and cloned in the pSB vectors (Kowarz et al., 2015) that are commercially available from Addgene (Addgene 60497, 60504, 60507 and 60526). The coding DNA sequences of all Kv7 constructs used in this study are provided in Dataset 1. Stable cell lines were established in HEK293T cells (American Type Culture Collection) using the pSB vectors as described previously (Wasano et al., 2020). A list of the cell lines generated in this study is provided in Table S1. The stable cell lines were cultured in Dulbecco's modified Eagle's medium (DMEM) (11965092, Thermo Fisher Scientific) supplemented with 10% fetal bovine serum (FBS; 10437028, Thermo Fisher Scientific) and penicillin-streptomycin (15140122, Thermo Fisher Scientific) at 37°C (5% CO_2_). Stable cell lines were also established in HEI-OC1 cells [a gift from Dr Federico Kalinac (Kalinac et al., 2003)], and in LLC-PK1 cells (a gift from Dr Tomohiro Shima, The University of Tokyo, Tokyo, Japan). HEI-OC1 stable cells were cultured in DMEM supplemented with 10% FBS at 33°C with 10% CO_2_, and LLC-PK1 stable cells were cultured in Medium 199 (11150059; Thermo Fisher Scientific) supplemented with 10% FBS and penicillin-streptomycin at 37°C (5% CO_2_).

### Electrophysiology

The electrophysiological properties of cell lines expressing various Kv7 constructs were determined at room temperature using an Axopatch 200B amplifier (Molecular Devices) in the whole-cell configuration. Recording pipettes were pulled from borosilicate glass to achieve initial bath resistances averaging 3 MΩ. Recording pipettes were filled with an intracellular solution containing 140 mM KCl, 2 mM MgCl_2_, 10 mM EGTA and 10 mM HEPES (pH 7.3). Cells were bathed in Hank's balanced salt solution (14025, Thermo Fisher Scientific). jClamp (SciSoft Company) was used to collect data. Expression of the Kv7 constructs was induced by application of doxycycline at 1 µg/ml (D9891, Sigma-Aldrich) to the cell culture medium 1 day before the experiments. Cell lines #001-014 (Table S1) were used for the whole-cell patch-clamp experiments.

### Co-precipitation assay

Cells co-expressing RFP-Kv7.4^WT^ and GFP-Kv7.4^WT^ or GFP-Kv7.4 variant were washed once with PBS and lysed in a buffer containing 150 mM NaCl, 20 mM n-dodecyl-β-D-maltoside (DDM), 1 mM EDTA, 1 mM dithiothreitol (DTT), 20 mM HEPES and 50 µg/ml leupeptin (pH 7.5). Supernatants were collected after centrifugation (13,000 ***g*** for 10 min at 4°C) and incubated with RFP-selector (N0410-L, NanoTag Biotechnologies) for 30 min at 4°C on a nutator. RFP-selector was collected by centrifugation (1000 ***g*** for 5 s at room temperature) and washed twice with a buffer containing 150 mM NaCl, 2 mM DDM, 1 mM EDTA, 1 mM DTT and 20 mM HEPES (pH 7.5). GFP-selector (N0310, NanoTag Biotechnologies) was added to the unbound fraction, incubated for 30 min at 4°C on a nutator, collected by centrifugation (1000 ***g*** for 5 s at room temperature) and washed twice with the wash buffer. The fluorescence of the RFP- and GFP-selector beads was imaged using an inverted epifluorescence microscope (DM IRB, Leica) equipped with a CMOS camera (ORCA-Flash4.0 V3, Hamamatsu). The obtained images were analyzed using Fiji (Schindelin et al., 2012). Cell lines #015-020 (Table S1) were used.

### Cell death assays

One day prior to experiments, cells were plated in a 96-well plate at 2×10^4^ cells per well and cultured overnight in DMEM supplemented with 10% FBS at 37°C in 5% CO_2_ (33°C in 10% CO_2_ for HEI-OC1 cells). On the next day, the 96-well plate was transferred to a plate reader equipped with temperature control and CO_2_ capabilities (Synergy Neo2, Biotek). Cell death assays were performed at 37°C and 5% CO_2_ (33°C in 10% CO_2_ for HEI-OC1 cells) using a CellTox Green Cytotoxicity Assay kit (G8741, Promega) and RealTime-Glo Annexin V Apoptosis Assay kit (JA1000, Promega) following the manufacturer's instructions. These plate reader-based assays were started immediately after applying doxycycline (0-10 µg/ml). Fluorescence (CellTox Green Cytotoxicity assay) or luminescence (Annexin V-based apoptosis assay) were monitored every 5 min for 72 h. Cell lines #002-007 and #021-036 (Table S1) were used.

### XBP1 splicing assay

Untransfected HEK293T cells and stable cell lines expressing the Kv7.4 constructs were seeded on 12-well plates and treated with 2 µg/ml tunicamycin (654380; MilliporeSigma), 300 nM thapsigargin (586005; MilliporeSigma) or 1 µg/ml doxycycline. After 6 h of treatment, cells were collected and processed using a Quick-RNA Miniprep Plus Kit (R1057, Zymo Research) for total RNA. cDNA synthesis was carried out with 1 µg of total RNA and random primers using SuperScriptIII Reverse Transcriptase (18080044, Thermo Fisher Scientific). PCR reactions were performed with 1 µl of cDNA and unspliced/spliced/total XBP1 universal primers (Yoon et al., 2019), using Taq DNA polymerase (M0273S, New England Biolabs). PCR products were resolved in Novex TBE gels (10%, EC6275BOX, Themo Fisher Scientific), stained with ethidium bromide (161-0433, Bio-Rad) and imaged using a Kodak Imaging System (Molecular Bioimaging). Band intensities were measured using Fiji (Schindelin et al., 2012).

### Drugs

The SCREEN-WELL Autophagy library containing 94 compounds was purchased from Enzo (BML-2837). FK-506 (tacrolimus) was purchased from Tocris (3631). SBI-0206965 was purchased from MilliporeSigma (SML1540). All autophagy-modulating compounds were reconstituted in dimethyl sulfoxide (DMSO), except chloroquine, which was reconstituted in water, and used at the final concentration of 10 µM. A caspase inhibitor, Z-VAD-FMK (219007, MilliporeSigma), and a necrosis inhibitor, necrostatin-1 (480065, MilliporeSigma), were reconstituted in DMSO and used at final concentrations of 20 µM and 40 µM, respectively.

### Statistical analyses

Statistical analyses were performed using Prism (GraphPad software). One-way ANOVA followed by the Tukey–Kramer test was used for multiple comparisons. A one-sample *t*-test was performed for evaluating the effects of drugs. In all these tests, *P*<0.05 was considered statistically significant.

## Supplementary Material

Supplementary information
