# Supplementary material for: Cell death-inducing cytotoxicity in truncated KCNQ4 variants associated with DFNA2 hearing loss
Source: Dis Model Mech. 2021 Nov 26;14(11):dmm049015. doi: 10.1242/dmm.049015 (PMC8628632; doi:10.1242/dmm.049015)
Supplement: Supplementary information [file dmm-14-049015-s1.pdf]

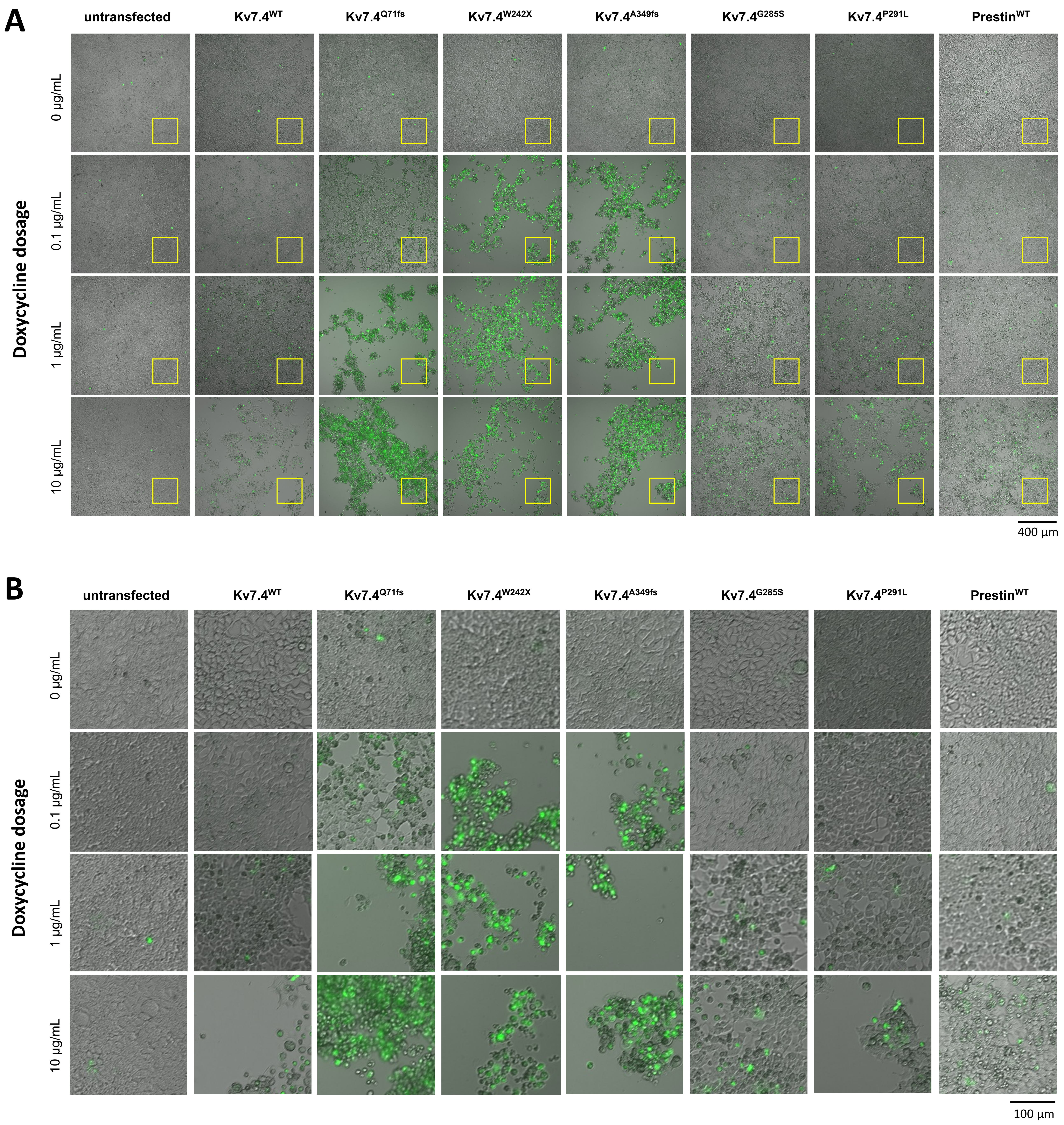


**Fig. S1.** High resolution images of cells after the CellTox Green Cytotoxicity assay (shown in Fig. 3C). Bright field and fluorescence images are merged in each panel. The regions of interest indicated by yellow boxes in panel A are magnified in panel B.

Disease Models & Mechanisms • Supplementary information

**Table S1**. Stable cell lines generated in this study.

| Stable cell line | Host cell | Construct(s) to be expressed | Vector(s) used | Selection |
|---|---|---|---|---|
| #001 | HEK293T | Kv7.4$^{WT}$ | pSBtet-RP | puromycin |
| #002 | | Kv7.4$^{WT}$ | pSBtet-GB | blasticidin |
| #003 | | Kv7.4$^{Q71fs}$ | pSBtet-GB | blasticidin |
| #004 | | Kv7.4$^{W242X}$ | pSBtet-GB | blasticidin |
| #005 | | Kv7.4$^{A349fs}$ | pSBtet-GB | blasticidin |
| #006 | | Kv7.4$^{G285S}$ | pSBtet-GB | blasticidin |
| #007 | | Kv7.4$^{P291L}$ | pSBtet-GB | blasticidin |
| #008 | | Kv7.4$^{WT}$<br>Kv7.4$^{WT}$ | pSBtet-RP<br>pSBtet-GB | puromycin<br>blasticidin |
| #009 | | Kv7.4$^{WT}$<br>Kv7.4$^{Q71fs}$ | pSBtet-RP<br>pSBtet-GB | puromycin<br>blasticidin |
| #010 | | Kv7.4$^{WT}$<br>Kv7.4$^{W242X}$ | pSBtet-RP<br>pSBtet-GB | puromycin<br>blasticidin |
| #011 | | Kv7.4$^{WT}$<br>Kv7.4$^{A349fs}$ | pSBtet-RP<br>pSBtet-GB | puromycin<br>blasticidin |
| #012 | | Kv7.4$^{WT}$-P2A-Kv7.4$^{Q71fs}$ | pSBtet-RP | puromycin |
| #013 | | Kv7.4$^{WT}$-P2A-Kv7.4$^{W242X}$ | pSBtet-RP | puromycin |
| #014 | | Kv7.4$^{WT}$-P2A-Kv7.4$^{A349fs}$ | pSBtet-RP | puromycin |
| #015 | | **RFP**-Kv7.4$^{WT}$<br>**GFP**-Kv7.4$^{WT}$ | pSBtet-Pur<br>pSBbi-Bla | puromycin<br>blasticidin |
| #016 | | **RFP**-Kv7.4$^{WT}$<br>**GFP**-Kv7.4$^{Q71fs}$ | pSBtet-Pur<br>pSBbi-Bla | puromycin<br>blasticidin |
| #017 | | **RFP**-Kv7.4$^{WT}$<br>**GFP**-Kv7.4$^{W242X}$ | pSBtet-Pur<br>pSBbi-Bla | puromycin<br>blasticidin |
| #018 | | **RFP**-Kv7.4$^{WT}$<br>**GFP**-Kv7.4$^{A349fs}$ | pSBtet-Pur<br>pSBbi-Bla | puromycin<br>blasticidin |
| #019 | | **RFP**-Kv7.4$^{WT}$<br>**GFP**-Kv7.4$^{G285S}$ | pSBtet-Pur<br>pSBbi-Bla | puromycin<br>blasticidin |
| #020 | | **RFP**-Kv7.4$^{WT}$<br>**GFP**-Kv7.4$^{P291L}$ | pSBtet-Pur<br>pSBbi-Bla | puromycin<br>blasticidin |
| #021 | | Kv7.1$^{WT}$ | pSBtet-RP | puromycin |
| #022 | | Kv7.1$^{E261X}$ | pSBtet-RP | puromycin |
| #023 | | Kv7.1$^{W305X}$ | pSBtet-RP | puromycin |
| #024 | | Kv7.1$^{Q530X}$ | pSBtet-RP | puromycin |
| #025 | | Kv7.1$^{Q531X}$ | pSBtet-RP | puromycin |
| #026 | | Prestin$^{WT}$ | pSBtet-GB | blasticidin |
| #027 | HEI-OC1 | Kv7.4$^{WT}$ | pSBtet-GB | blasticidin |
| #028 | | Kv7.4$^{Q71fs}$ | pSBtet-GB | blasticidin |
| #029 | | Kv7.4$^{W242X}$ | pSBtet-GB | blasticidin |
| #030 | | Kv7.4$^{A349fs}$ | pSBtet-GB | blasticidin |
| #031 | | Prestin$^{WT}$ | pSBtet-GB | blasticidin |
| #032 | LLC-PK1 | Kv7.4$^{WT}$ | pSBtet-GB | blasticidin |
| #033 | | Kv7.4$^{Q71fs}$ | pSBtet-GB | blasticidin |
| #034 | | Kv7.4$^{W242X}$ | pSBtet-GB | blasticidin |
| #035 | | Kv7.4$^{A349fs}$ | pSBtet-GB | blasticidin |
| #036 | | Prestin$^{WT}$ | pSBtet-GB | blasticidin |

**Dataset 1. The cDNA sequences of the Kv7.4(KCNQ4) and Kv7.1(KCNQ1) constructs used in this study**

**Gray highlights** indicate stop codons.
**Yellow highlights** indicate frame-shifted coding sequences.
**Red highlights** indicate missense changes.

**Kv7.4$^{WT}$** (human wild-type)
ATGGCCGAGGCCCCCCCGCGCCGCCTCGGCCTGGGTCCCCCGCCCGGGGACGCCCCCCGCGCGGAGCTAGTGGCGC
TCACGGCCGTGCAGAGCGAACAGGGCGAGGCGGGCGGGGGCGGCTCCCCGCGCCGCCTCGGCCTCCTGGGCAGCCC
CCTGCCGCCGGGCGCGCCCCTCCCTGGGCCGGGCTCCGGCTCGGGCTCCGCCTGCGGCCAGCGCTCCTCGGCCGCG
CACAAGCGCTACCGCCGCCTGCAGAACTGGGTCTACAACGTGCTGGAGCGGCCCCGCGGCTGGGCCTTCGTCTACC
ACGTCTTCATATTTTTGCTGGTCTTCAGCTGTCTGGTGCTGTCTGTGCTGTCCACTATCCAGGAGCACCAGGAACT
TGCCAACGAGTGTCTCCTCATCTTGGAATTCGTGATGATCGTGGTTTTCGGCTTGGAGTACATCGTCCGGGTCTGG
TCCGCCGGATGCTGCTGCCGCTACCGAGGATGGCAGGGTCGCTTCCGCTTTGCCAGAAAGCCCTTCTGTGTCATCG
ACTTCATCGTGTTCGTGGCCTCGGTGGCCGTCATCGCCGCGGGTACCCAGGGCAACATCTTCGCCACGTCCGCGCT
GCGCAGCATGCGCTTCCTGCAGATCCTGCGCATGGTGCGCATGGACCGCCGCGGCGGCACCTGGAAGCTGCTGGGC
TCAGTGGTCTACGCGCATAGCAAGGAGCTGATCACCGCCTGGTACATCGGGTTCCTGGTGCTCATCTTCGCCTCCT
TCCTGGTCTACCTGGCCGAGAAGGACGCCAACTCCGACTTCTCCTCCTACGCCGACTCGCTCTGGTGGGGGACGAT
TACATTGACAACCATCGGCTATGGTGACAAGACACCGCACACATGGCTGGGCAGGGTCCTGGCTGCTGGCTTCGCC
TTACTGGGCATCTCTTTCTTTGCCCTGCCTGCCGGCATCCTAGGCTCCGGCTTTGCCCTGAAGGTCCAGGAGCAGC
ACCGGCAGAAGCACTTCGAGAAGCGGAGGATGCCGGCAGCCAACCTCATCCAGGCTGCCTGGCGCCTGTACTCCAC
CGATATGAGCCGGGCCTACCTGACAGCCACCTGGTACTACTATGACAGTATCCTCCCATCCTTCAGAGAGCTGGCC
CTCTTGTTTGAGCACGTGCAACGGGCCCGCAATGGGGGCCTACGGCCCCTGGAGGTGCGGCGGGCGCCGGTACCCG
ACGGAGCACCCTCCCGTTACCCGCCCGTTGCCACCTGCCACCGGCCGGGCAGCACCTCCTTCTGCCCTGGGAAAG
CAGCCGGATGGGCATCAAAGACCGCATCCGCATGGGCAGCTCCCAGCGGCGGACGGGTCCTTCCAAGCAGCATCTG
GCACCTCCAACAATGCCCACCTCCCCAAGCAGCGAGCAGGTGGGTGAGGCCACCAGCCCCACCAAGGTGCAAAAGA
GCTGGAGCTTCAATGACCGCACCCGCTTCCGGGCATCTCTGAGACTCAAACCCCGCACCTCTGCTGAGGATGCCCC
CTCAGAGGAAGTAGCAGAGGAGAAGAGCTACCAGTGTGAGCTCACGGTGGACGACATCATGCCTGCTGTGAAGACA
GTCATCCGCTCCATCAGGATTCTCAAGTTCCTGGTGGCCAAAAGGAAATTCAAGGAGACACTGCGACCGTACGACG
TGAAGGACGTCATTGAGCAGTACTCAGCAGGCCACCTGGACATGCTGGGCCGGATCAAGAGCCTGCAAACTCGGGT
GGACCAAATTGTGGGTCGGGGGCCCGGGGACAGGAAGGCCCGGGAGAAGGGCGACAAGGGGCCCTCCGACGCGGAG
GTGGTGGATGAAATCAGCATGATGGGACGCGTGGTCAAGGTGGAGAAGCAGGTGCAGTCCATCGAGCACAAGCTGG
ACCTGCTGTTGGGCTTCTATTCGCGCTGCCTGCGCTCTGGCACCTCGGCCAGCCTGGGCGCCGTGCAAGTGCCGCT
GTTCGACCCCGACATCACCTCCGACTACCACAGCCCTGTGGACCACGAGGACATCTCCGTCTCCGCACAGACGCTC
AGCATCTCCCGCTCGGTCAGCACCAACATGGACTGA

**Kv7.4$^{Q71fs}$**(c.211delC)
ATGGCCGAGGCCCCCCCGCGCCGCCTCGGCCTGGGTCCCCCGCCCGGGGACGCCCCCCGCGCGGAGCTAGTGGCGC
TCACGGCCGTGCAGAGCGAACAGGGCGAGGCGGGCGGGGGCGGCTCCCCGCGCCGCCTCGGCCTCCTGGGCAGCCC
CCTGCCGCCGGGCGCGCCCCTCCCTGGGCCGGGCTCCGGCTCGGGCTCCGCCTGCGGCAGCGCTCCTCGGCCGCGC
ACAAGCGCTACCGCCGCCTGCAGAACTGGGTCTACAACGTGCTGGAGCGGCCCCGCGGCTGGGCCTTCGTCTACCA
CGTCTTCATATTTTTGCTGGTCTTCAGCTGTCTGGTGCTGTCTGTGCTGTCCACTATCCAGGAGCACCAGGAACTT
GCCAACGAGTGTCTCCTCATCTTGGAATTCGTGA

**Kv7.4$^{W242X}$**(c.725G>A)
ATGGCCGAGGCCCCCCCGCGCCGCCTCGGCCTGGGCCCCCCGCCCGGGGACGCCCCCCGCGCGGAGTTGGTGGCGC
TCACGGCCGTGCAGAGTGAACAGGGCGAGGCGGGCGGGGGCGGCTCTCCGCGTCGCCTCGGCCTTCTGGGCAGCCC
CCTGCCGCCGGGCGCGCCCCTCCCTGGGCCGGGCTCCGGCTCGGGCTCCGCCTGCGGCGGCcAGCGCTCCTCCGCC
GCGCAGAAGCGCTACCGCCGCCTGCAGAACTGGGTCTACAACGTGCTGGAGCGGCCCCGCGGGTGGGCCTTCGTCT
ACCACGTCTTCATATTTTTGCTAGTCTTCAGCTGCCTGGTGCTGTCTGTACTGTCCACCATCCAGGAGCACCAGGA
ACTTGCCAACGAGTGTCTCCTTATCTTGGAATTCGTGATGATTGTGGTCTTTGGCTTGGAGTATATAATCCGTGTC
TGGTCGGCCGGATGCTGTTGTCGCTACAGAGGATGGCAGGGACGCTTTCGCTTCGCCAGGAAACCCTTCTGTGTCA
TCGACTTCATCGTGTTCGTGGCCTCGGTGGCAGTGATAGCTGCGGGCACACAAGGCAACATCTTTGCTACGTCCGC
GTTGCGCAGTATGCGCTTCCTACAGATCCTGCGCATGGTGCGTATGGATCGCCGCGGTGGCACCTGGAAGCTGTTG
GGATCCGTGGTCTATGCGCACAGTAAGGAGCTGATCACCGCCTAG

Disease Models & Mechanisms • Supplementary information

**Kv7.4**$^{A349fs}$ (c.1044_1051del8)
ATGGCCGAGGCCCCCCCGCGCCGCCTCGGCCTGGGTCCCCCGCCCGGGGACGCCCCCCGCGCGGAGCTAGTGGCGC
TCACGGCCGTGCAGAGCGAACAGGGCGAGGCGGGCGGGGGCGGCTCCCCGCGCCGCCTCGGCCTCCTGGGCAGCCC
CCTGCCGCCGGGCGCGCCCCTCCCTGGGCCGGGCTCCGGCTCGGGCTCCGCCTGCGGCCAGCGCTCCTCGGCCGCG
CACAAGCGCTACCGCCGCCTGCAGAACTGGGTCTACAACGTGCTGGAGCGGCCCCGCGGCTGGGCCTTCGTCTACC
ACGTCTTCATATTTTTGCTGGTCTTCAGCTGTCTGGTGCTGTCTGTGCTGTCCACTATCCAGGAGCACCAGGAACT
TGCCAACGAGTGTCTCCTCATCTTGGAATTCGTGATGATCGTGGTTTTCGGCTTGGAGTACATCGTCCGGGTCTGG
TCCGCCGGATGCTGCTGCCGCTACCGAGGATGGCAGGGTCGCTTCCGCTTTGCCAGAAAGCCCTTCTGTGTCATCG
ACTTCATCGTGTTCGTGGCCTCGGTGGCCGTCATCGCCGCGGGTACCCAGGGCAACATCTTCGCCACGTCCGCGCT
GCGCAGCATGCGCTTCCTGCAGATCCTGCGCATGGTGCGCATGGACCGCCGCGGCGGCACCTGGAAGCTGCTGGGC
TCAGTGGTCTACGCGCATAGCAAGGAGCTGATCACCGCCTGGTACATCGGGTTCCTGGTGCTCATCTTCGCCTCCT
TCCTGGTCTACCTGGCCGAGAAGGACGCCAACTCCGACTTCTCCTCCTACGCCGACTCGCTCTGGTGGGGGACGAT
TACATTGACAACCATCGGCTATGGTGACAAGACACCGCACACATGGCTGGGCAGGGTCCTGGCTGCTGGCTTCGCC
TTACTGGGCATCTCTTTCTTTGCCCTGCCTGCCGGCATCCTAGGCTCCGGCTTTGCCCTGAAGGTCCAGGAGCAGC
ACCGGCAGAAGCACTTCGAGAAGCGGAGGATGCCGGCAGCCAACCTCATCCAGGCGCCTGTACTCCACCGATATGA
GCCGGGCCTACCTGACAGCCACCTGGTACTACTATGA

**Kv7.4**$^{G285S}$(c.853G>A)
ATGGCCGAGGCCCCCCCGCGCCGCCTCGGCCTGGGTCCCCCGCCCGGGGACGCCCCCCGCGCGGAGCTAGTGGCGC
TCACGGCCGTGCAGAGCGAACAGGGCGAGGCGGGCGGGGGCGGCTCCCCGCGCCGCCTCGGCCtCCTGGGCAGCCC
CCTGCCGCCGGGCGCGCCCCTCCCTGGGCCGGGCTCCGGCTCGGGCTCCGCCTGCGGCcAGCGCTCCTCGGCCGCG
CACAAGCGCTACCGCCGCCTGCAGAACTGGGTCTACAACGTGCTGGAGCGGCCCCGCGGCTGGGCCTTCGTCTACC
ACGTCTTCATATTTTTGCTGGTCTTCAGCTGTCTGGTGCTGTCTGTGCTGTCCACTATCCAGGAGCACCAGGAACT
TGCCAACGAGTGTCTCCTCATCTTGGAATTCGTGATGATCGTGGTTTTCGGCTTGGAGTACATCGTCCGGGTCTGG
TCCGCCGGATGCTGCTGCCGCTACCGAGGATGGCAGGGTCGCTTCCGCTTTGCCAGAAAGCCCTTCTGTGTCATCG
ACTTCATCGTGTTCGTGGCCTCGGTGGCCGTCATCGCCGCGGGTACCCAGGGCAACATCTTCGCCACGTCCGCGCT
GCGCAGCATGCGCTTCCTGCAGATCCTGCGCATGGTGCGCATGGACCGCCGCGGCGGCACCTGGAAGCTGCTGGGC
TCAGtGGTCTACGCGCaTAGCAAGGAGCTGATCACCGCCTGGTACATCGGGTTCCTGGTGCTCATCTTCgCCTCCT
TCCTGGTCTACCTGGCCGAGAAGGACGCCAACTCCGACTTCTCCTcctACGCCGACTCGCTCTGGTgGGGGACGAT
TACATTGACAACCATCAGCTATGGTGACAAGACACcGCACACATGGCTGGGCAGGGTCCTGGCTGCTGGCTTCGCC
TTACTGGGCATCTCTTTCTTTGCCCTGCCTGCCGGCATCCTAGGCTCCGGCTTTGCCCTGAAGGTCCAGGAGCAGC
ACCGGCAGAAGCACTTCGAGAAGCGGAGGATGCCGGCAGCCAACCTCATCCAGGCTGCCTGGCGCCTGTACTCCAC
CGATATGAGCCGGGCCTACCTGACAGCCACCTGGTACTACTATGACAGTATCCTCCCATCCTTCAGAGAGCTGGCC
CTCTTGTTTGAGCACGTGCAACGGGCCCGCAATGGGGGCCTACGGCCCCTGGAGGTGCGGCGGGCGCCGGTACCCG
ACGGAGCACCCTCCCGTTACCCGCCCGTTGCCACCTGCCACCGGCCGGGCAGCACCTCCTTCTGCCCTGGGGAAAG
CAGCCGGATGGGCATCAAAGACCGCATCCGCATGGGCAGCTCCCAGCGGCGGACGGGTCCTTCCAAGCAGCATCTG
GCACCTCCAACAATGCCCACCTCCCCAAGCAGCGAGCAGGTGGGTGAGGCCACCAGCCCCACCAAGGTGCAAAAGA
GCTGGAGCTTCAATGACCGCACCCGCTTCCGGGCATCTCTGAGACTCAAACCCCGCACCTCTGCTGAGGATGCCCC
CTCAGAGGAAGTAGCAGAGGAGAAGAGCTACCAGTGTGAGCTCACGGTGGACGACATCATGCCTGCTGTGAAGACA
GTCATCCGCTCCATCAGGATTCTCAAGTTCCTGGTGGCCAAAAGGAAATTCAAGGAGACACTGCGACCGTACGACG
TGAAGGACGTCATTGAGCAGTACTCAGCAGGCCACCTGGACATGCTGGGCCGgATCAAGAGCCTGCAAACTCGGGT
GGACCAAATTGTGGGTCGGGGGCCCGGGGACAGGAAGGCCCGGGAGAAGGGCGACAAGGGGCCCTCCGACGCGGAG
GTGGTGGATGAAATCAGCATGATGGGACGCGTGGTCAAGGTGGAGAAGCAGGTGCAGTCCATCGAGCACAAGCTGG
ACCTGCTGTTGGGCTTCTATTCGCGCTGCCTGCGCTCTGGCACCTCGGCCAGCCTGGGCGCCGTGCAAGTGCCGCT
GTTCGACCCCGACATCACCTCCGACTACCACAGCCCTGTGGACCACGAGGACATCTCCGTCTCCGCACAGACGCTC
AGCATCTCCCGCTCGGTCAGCACCAACATGGACTGA

**Kv7.4**$^{P291L}$ (c.872C>T)
ATGGCCGAGGCCCCCCCGCGCCGCCTCGGCCTGGGTCCCCCGCCCGGGGACGCCCCCCGCGCGGAGCTAGTGGCGC
TCACGGCCGTGCAGAGCGAACAGGGCGAGGCGGGCGGGGGCGGCTCCCCGCGCCGCCTCGGCCtCCTGGGCAGCCC
CCTGCCGCCGGGCGCGCCCCTCCCTGGGCCGGGCTCCGGCTCGGGCTCCGCCTGCGGCcAGCGCTCCTCGGCCGCG
CACAAGCGCTACCGCCGCCTGCAGAACTGGGTCTACAACGTGCTGGAGCGGCCCCGCGGCTGGGCCTTCGTCTACC
ACGTCTTCATATTTTTGCTGGTCTTCAGCTGTCTGGTGCTGTCTGTGCTGTCCACTATCCAGGAGCACCAGGAACT
TGCCAACGAGTGTCTCCTCATCTTGGAATTCGTGATGATCGTGGTTTTCGGCTTGGAGTACATCGTCCGGGTCTGG
TCCGCCGGATGCTGCTGCCGCTACCGAGGATGGCAGGGTCGCTTCCGCTTTGCCAGAAAGCCCTTCTGTGTCATCG

Disease Models & Mechanisms • Supplementary information

ACTTCATCGTGTTCGTGGCCTCGGTGGCCGTCATCGCCGCGGGTACCCAGGGCAACATCTTCGCCACGTCCGCGCT
GCGCAGCATGCGCTTCCTGCAGATCCTGCGCATGGTGCGCATGGACCGCCGCGGCGGCACCTGGAAGCTGCTGGGC
TCAGtGGTCTACGCGCaTAGCAAGGAGCTGATCACCGCCTGGTACATCGGGTTCCTGGTGCTCATCTTCgCCTCCT
TCCTGGTCTACCTGGCCGAGAAGGACGCCAACTCCGACTTCTCCTcctACGCCGACTCGCTCTGGTgGGGACGAT
TACATTGACAACCATCgGCTATGGTGACAAGACACTGCACACATGGCTGGGCAGGGTCCTGGCTGCTGGCTTCGCC
TTACTGGGCATCTCTTTCTTTGCCCTGCCTGCCGGCATCCTAGGCTCCGGCTTTGCCCTGAAGGTCCAGGAGCAGC
ACCGGCAGAAGCACTTCGAGAAGCGGAGGATGCCGGCAGCCAACCTCATCCAGGCTGCCTGGCGCCTGTACTCCAC
CGATATGAGCCGGGCCTACCTGACAGCCACCTGGTACTACTATGACAGTATCCTCCCATCCTTCAGAGAGCTGGCC
CTCTTGTTTGAGCACGTGCAACGGGCCCGCAATGGGGGCCTACGGCCCCTGGAGGTGCGGCGGGCGCCGGTACCCG
ACGGAGCACCCTCCCGTTACCCGCCCGTTGCCACCTGCCACCGGCCGGGCAGCACCTCCTTCTGCCCTGGGGAAAG
CAGCCGGATGGGCATCAAAGACCGCATCCGCATGGGCAGCTCCCAGCGGCGGACGGGTCCTTCCAAGCAGCATCTG
GCACCTCCAACAATGCCCACCTCCCCAAGCAGCGAGCAGGTGGGTGAGGCCACCAGCCCCACCAAGGTGCAAAAGA
GCTGGAGCTTCAATGACCGCACCCGCTTCCGGGCATCTCTGAGACTCAAACCCCGCACCTCTGCTGAGGATGCCCC
CTCAGAGGAAGTAGCAGAGGAGAAGAGCTACCAGTGTGAGCTCACGGTGGACGACATCATGCCTGCTGTGAAGACA
GTCATCCGCTCCATCAGGATTCTCAAGTTCCTGGTGGCCAAAAGGAAATTCAAGGAGACACTGCGACCGTACGACG
TGAAGGACGTCATTGAGCAGTACTCAGCAGGCCACCTGGACATGCTGGGCCGgATCAAGAGCCTGCAAACTCGGGT
GGACCAAATTGTGGGTCGGGGGCCCGGGGACAGGAAGGCCCGGGAGAAGGGCGACAAGGGGCCCTCCGACGCGGAG
GTGGTGGATGAAATCAGCATGATGGGACGCGTGGTCAAGGTGGAGAAGCAGGTGCAGTCCATCGAGCACAAGCTGG
ACCTGCTGTTGGGCTTCTATTCGCGCTGCCTGCGCTCTGGCACCTCGGCCAGCCTGGGCGCCGTGCAAGTGCCGCT
GTTCGACCCCGACATCACCTCCGACTACCACAGCCCTGTGGACCACGAGGACATCTCCGTCTCCGCACAGACGCTC
AGCATCTCCCGCTCGGTCAGCACCAACATGGACTGA

**RFP-Kv7.4<sup>WT</sup>(RFP-linker-Kv7.4<sup>WT</sup>)**

ATGGCCTCCTCCGAGGACGTCATCAAGGAGTTCATGCGCTTCAAGGTGCGCATGGAGGGCTCCGTGAACGGCCACG
AGTTCGAGATCGAGGGCGAGGGCGAGGGCCGCCCCTACGAGGGCACCCAGACCGCCAAGCTGAAGGTGACCAAGGG
CGGCCCCCTGCCCTTCGCCTGGGACATCCTGTCCCCTCAGTTCCAGTACGGCTCCAAGGCCTACGTGAAGCACCCC
GCCGACATCCCCGACTACTTGAAGCTGTCCTTCCCCGAGGGCTTCAAGTGGGAGCGCGTGATGAACTTCGAGGACG
GCGGCGTGGTGACCGTGACCCAGGACTCCTCCCTGCAGGACGGCGAGTTCATCTACAAGGTGAAGCTGCGCGGCAC
CAACTTCCCCTCCGACGGCCCCGTAATGCAGAAGAAGACCATGGGCTGGGAGGCCTCCACCGAGCGGATGTACCCC
GAGGACGGCGCCCTGAAGGGCGAGATCAAGATGAGGCTGAAGCTGAAGGACGGCGGCCACTACGACGCCGAGGTCA
AGACCACCTACATGGCCAAGAAGCCCGTGCAGCTGCCCGGCGCCTACAAGACCGACATCAAGCTGGACATCACCTC
CCACAACGAGGACTACACCATCGTGGAACAGTACGAGCGCGCCGAGGGCCGCCACTCCACCGGCGCCCTGTACAAG
TCCGGACTCAGATCTCGAGCTCAAGCTTCGAATTCTGCAGTCGACGGTACCGCGGGCCCGGGATCCACCATGGCCG
AGGCCCCCCCGCGCCGCCTCGGCCTGGGTCCCCCGCCCGGGGACGCCCCCCGCGCGGAGCTAGTGGCGCTCACGGC
CGTGCAGAGCGAACAGGGCGAGGCGGGCGGGGGCGGCTCCCCGCGCCGCCTCGGCCTCCTGGGCAGCCCCCTGCCG
CCGGGCGCGCCCCTCCCTGGGCCGGGCTCCGGCTCGGGCTCCGCCTGCGGCCAGCGCTCCTCGGCCGCGCACAAGC
GCTACCGCCGCCTGCAGAACTGGGTCTACAACGTGCTGGAGCGGCCCCGCGGCTGGGCCTTCGTCTACCACGTCTT
CATATTTTTGCTGGTCTTCAGCTGTCTGGTGCTGTCTGTGCTGTCCACTATCCAGGAGCACCAGGAACTTGCCAAC
GAGTGTCTCCTCATCTTGGAATTCGTGATGATCGTGGTTTTCGGCTTGGAGTACATCGTCCGGGTCTGGTCCGCCG
GATGCTGCTGCCGCTACCGAGGATGGCAGGGTCGCTTCCGCTTTGCCAGAAAGCCCTTCTGTGTCATCGACTTCAT
CGTGTTCGTGGCCTCGGTGGCCGTCATCGCCGCGGGTACCCAGGGCAACATCTTCGCCACGTCCGCGCTGCGCAGC
ATGCGCTTCCTGCAGATCCTGCGCATGGTGCGCATGGACCGCCGCGGCGGCACCTGGAAGCTGCTGGGCTCAGTGG
TCTACGCGCATAGCAAGGAGCTGATCACCGCCTGGTACATCGGGTTCCTGGTGCTCATCTTCGCCTCCTTCCTGGT
CTACCTGGCCGAGAAGGACGCCAACTCCGACTTCTCCTCCTACGCCGACTCGCTCTGGTGGGGGACGATTACATTG
ACAACCATCGGCTATGGTGACAAGACACCGCACACATGGCTGGGCAGGGTCCTGGCTGCTGGCTTCGCCTTACTGG
GCATCTCTTTCTTTGCCCTGCCTGCCGGCATCCTAGGCTCCGGCTTTGCCCTGAAGGTCCAGGAGCAGCACCGGCA
GAAGCACTTCGAGAAGCGGAGGATGCCGGCAGCCAACCTCATCCAGGCTGCCTGGCGCCTGTACTCCACCGATATG
AGCCGGGCCTACCTGACAGCCACCTGGTACTACTATGACAGTATCCTCCCATCCTTCAGAGAGCTGGCCCTCTTGT
TTGAGCACGTGCAACGGGCCCGCAATGGGGGCCTACGGCCCCTGGAGGTGCGGCGGGCGCCGGTACCCGACGGAGC
ACCCTCCCGTTACCCGCCCGTTGCCACCTGCCACCGGCCGGGCAGCACCTCCTTCTGCCCTGGGGAAAGCAGCCGG
ATGGGCATCAAAGACCGCATCCGCATGGGCAGCTCCCAGCGGCGGACGGGTCCTTCCAAGCAGCATCTGGCACCTC
CAACAATGCCCACCTCCCCAAGCAGCGAGCAGGTGGGTGAGGCCACCAGCCCCACCAAGGTGCAAAAGAGCTGGAG
CTTCAATGACCGCACCCGCTTCCGGGCATCTCTGAGACTCAAACCCCGCACCTCTGCTGAGGATGCCCCCTCAGAG
GAAGTAGCAGAGGAGAAGAGCTACCAGTGTGAGCTCACGGTGGACGACATCATGCCTGCTGTGAAGACAGTCATCC
GCTCCATCAGGATTCTCAAGTTCCTGGTGGCCAAAAGGAAATTCAAGGAGACACTGCGACCGTACGACGTGAAGGA
CGTCATTGAGCAGTACTCAGCAGGCCACCTGGACATGCTGGGCCGGATCAAGAGCCTGCAAACTCGGGTGGACCAA

Disease Models & Mechanisms • Supplementary information

ATTGTGGGTCGGGGGCCCGGGGACAGGAAGGCCCGGGAGAAGGGCGACAAGGGGCCCTCCGACGCGGAGGTGGTGG
ATGAAATCAGCATGATGGGACGCGTGGTCAAGGTGGAGAAGCAGGTGCAGTCCATCGAGCACAAGCTGGACCTGCT
GTTGGGCTTCTATTCGCGCTGCCTGCGCTCTGGCACCTCGGCCAGCCTGGGCGCCGTGCAAGTGCCGCTGTTCGAC
CCCGACATCACCTCCGACTACCACAGCCCTGTGGACCACGAGGACATCTCCGTCTCCGCACAGACGCTCAGCATCT
CCCGCTCGGTCAGCACCAACATGGACTGA

**GFP-Kv7.4$^{WT}$(GFP-linker-Kv7.4$^{WT}$)**

ATGGTGAGCAAGGGCGAGGAGCTGTTCACCGGGGTGGTGCCCATCCTGGTCGAGCTGGACGGCGACGTAAACGGCC
ACAAGTTCAGCGTGTCCGGCGAGGGCGAGGGCGATGCCACCTACGGCAAGCTGACCCTGAAGTTCATCTGCACCAC
CGGCAAGCTGCCCGTGCCCTGGCCCACCCTCGTGACCACCCTGACCTACGGCGTGCAGTGCTTCAGCCGCTACCCC
GACCACATGAAGCAGCACGACTTCTTCAAGTCCGCCATGCCCGAAGGCTACGTCCAGGAGCGCACCATCTTCTTCA
AGGACGACGGCAACTACAAGACCCGCGCCGAGGTGAAGTTCGAGGGCGACACCCTGGTGAACCGCATCGAGCTGAA
GGGCATCGACTTCAAGGAGGACGGCAACATCCTGGGGCACAAGCTGGAGTACAACTACAACAGCCACAACGTCTAT
ATCATGGCCGACAAGCAGAAGAACGGCATCAAGGTGAACTTCAAGATCCGCCACAACATCGAGGACGGCAGCGTGC
AGCTCGCCGACCACTACCAGCAGAACACCCCCATCGGCGACGGCCCCGTGCTGCTGCCCGACAACCACTACCTGAG
CACCCAGTCCGCCCTGAGCAAAGACCCCAACGAGAAGCGCGATCACATGGTCCTGCTGGAGTTCGTGACCGCCGCC
GGGATCACTCTCGGCATGGACGAGCTGTACAAGTCCGGACTCAGATCTCGAGCTCAAGCTTCGAATTCTGCAGTCG
ACGGTACCGCGGGCCCGGGATCCACCATGGCCGAGGCCCCCCCGCGCCGCCTCGGCCTGGGTCCCCCGCCCGGGGA
CGCCCCCCGCGCGGAGCTAGTGGCGCTCACGGCCGTGCAGAGCGAACAGGGCGAGGCGGGCGGGGGCGGCTCCCCG
CGCCGCCTCGGCCTCCTGGGCAGCCCCCTGCCGCCGGGCGCGCCCCTCCCTGGGCCGGGCTCCGGCTCGGGCTCCG
CCTGCGGCCAGCGCTCCTCGGCCGCGCACAAGCGCTACCGCCGCCTGCAGAACTGGGTCTACAACGTGCTGGAGCG
GCCCCGCGGCTGGGCCTTCGTCTACCACGTCTTCATATTTTTGCTGGTCTTCAGCTGTCTGGTGCTGTCTGTGCTG
TCCACTATCCAGGAGCACCAGGAACTTGCCAACGAGTGTCTCCTCATCTTGGAATTCGTGATGATCGTGGTTTTCG
GCTTGGAGTACATCGTCCGGGTCTGGTCCGCCGGATGCTGCTGCCGCTACCGAGGATGGCAGGGTCGCTTCCGCTT
TGCCAGAAAGCCCTTCTGTGTCATCGACTTCATCGTGTTCGTGGCCTCGGTGGCCGTCATCGCCGCGGGTACCCAG
GGCAACATCTTCGCCACGTCCGCGCTGCGCAGCATGCGCTTCCTGCAGATCCTGCGCATGGTGCGCATGGACCGCC
GCGGCGGCACCTGGAAGCTGCTGGGCTCAGTGGTCTACGCGCATAGCAAGGAGCTGATCACCGCCTGGTACATCGG
GTTCCTGGTGCTCATCTTCGCCTCCTTCCTGGTCTACCTGGCCGAGAAGGACGCCAACTCCGACTTCTCCTCCTAC
GCCGACTCGCTCTGGTGGGGGACGATTACATTGACAACCATCGGCTATGGTGACAAGACACCGCACACATGGCTGG
GCAGGGTCCTGGCTGCTGGCTTCGCCTTACTGGGCATCTCTTTCTTTGCCCTGCCTGCCGGCATCCTAGGCTCCGG
CTTTGCCCTGAAGGTCCAGGAGCAGCACCGGCAGAAGCACTTCGAGAAGCGGAGGATGCCGGCAGCCAACCTCATC
CAGGCTGCCTGGCGCCTGTACTCCACCGATATGAGCCGGGCCTACCTGACAGCCACCTGGTACTACTATGACAGTA
TCCTCCCATCCTTCAGAGAGCTGGCCCTCTTGTTTGAGCACGTGCAACGGGCCCGCAATGGGGGCCTACGGCCCCT
GGAGGTGCGGCGGGCGCCGGTACCCGACGGAGCACCCTCCCGTTACCCGCCCGTTGCCACCTGCCACCGGCCGGGC
AGCACCTCCTTCTGCCCTGGGGAAAGCAGCCGGATGGGCATCAAAGACCGCATCCGCATGGGCAGCTCCCAGCGGC
GGACGGGTCCTTCCAAGCAGCATCTGGCACCTCCAACAATGCCCACCTCCCCAAGCAGCGAGCAGGTGGGTGAGGC
CACCAGCCCCACCAAGGTGCAAAAGAGCTGGAGCTTCAATGACCGCACCCGCTTCCGGGCATCTCTGAGACTCAAA
CCCCGCACCTCTGCTGAGGATGCCCCCTCAGAGGAAGTAGCAGAGGAGAAGAGCTACCAGTGTGAGCTCACGGTGG
ACGACATCATGCCTGCTGTGAAGACAGTCATCCGCTCCATCAGGATTCTCAAGTTCCTGGTGGCCAAAAGGAAATT
CAAGGAGACACTGCGACCGTACGACGTGAAGGACGTCATTGAGCAGTACTCAGCAGGCCACCTGGACATGCTGGGC
CGGATCAAGAGCCTGCAAACTCGGGTGGACCAAATTGTGGGTCGGGGGCCCGGGGACAGGAAGGCCCGGGAGAAGG
GCGACAAGGGGCCCTCCGACGCGGAGGTGGTGGATGAAATCAGCATGATGGGACGCGTGGTCAAGGTGGAGAAGCA
GGTGCAGTCCATCGAGCACAAGCTGGACCTGCTGTTGGGCTTCTATTCGCGCTGCCTGCGCTCTGGCACCTCGGCC
AGCCTGGGCGCCGTGCAAGTGCCGCTGTTCGACCCCGACATCACCTCCGACTACCACAGCCCTGTGGACCACGAGG
ACATCTCCGTCTCCGCACAGACGCTCAGCATCTCCCGCTCGGTCAGCACCAACATGGACTGA

**GFP-Kv7.4$^{Q71fs}$(GFP-linker-Kv7.4$^{Q71fs}$)**

ATGGTGAGCAAGGGCGAGGAGCTGTTCACCGGGGTGGTGCCCATCCTGGTCGAGCTGGACGGCGACGTAAACGGCC
ACAAGTTCAGCGTGTCCGGCGAGGGCGAGGGCGATGCCACCTACGGCAAGCTGACCCTGAAGTTCATCTGCACCAC
CGGCAAGCTGCCCGTGCCCTGGCCCACCCTCGTGACCACCCTGACCTACGGCGTGCAGTGCTTCAGCCGCTACCCC
GACCACATGAAGCAGCACGACTTCTTCAAGTCCGCCATGCCCGAAGGCTACGTCCAGGAGCGCACCATCTTCTTCA
AGGACGACGGCAACTACAAGACCCGCGCCGAGGTGAAGTTCGAGGGCGACACCCTGGTGAACCGCATCGAGCTGAA
GGGCATCGACTTCAAGGAGGACGGCAACATCCTGGGGCACAAGCTGGAGTACAACTACAACAGCCACAACGTCTAT
ATCATGGCCGACAAGCAGAAGAACGGCATCAAGGTGAACTTCAAGATCCGCCACAACATCGAGGACGGCAGCGTGC
AGCTCGCCGACCACTACCAGCAGAACACCCCCATCGGCGACGGCCCCGTGCTGCTGCCCGACAACCACTACCTGAG
CACCCAGTCCGCCCTGAGCAAAGACCCCAACGAGAAGCGCGATCACATGGTCCTGCTGGAGTTCGTGACCGCCGCC

GGGATCACTCTCGGCATGGACGAGCTGTACAAGTCCGGACTCAGATCTCGAGCTCAAGCTTCGAATTCTGCAGTCG
ACGGTACCGCGGGCCCGGGATCCACCATGGCCGAGGCCCCCCCGCGCCGCCTCGGCCTGGGTCCCCCGCCCGGGGA
CGCCCCCCGCGCGGAGCTAGTGGCGCTCACGGCCGTGCAGAGCGAACAGGGCGAGGCGGGCGGGGGCGGCTCCCCG
CGCCGCCTCGGCCTCCTGGGCAGCCCCCTGCCGCCGGGCGCGCCCCTCCCTGGGCCGGGCTCCGGCTCGGGCTCCG
CCTGCGGCAGCGCTCCTCGGCCGCGCACAAGCGCTACCGCCGCCTGCAGAACTGGGTCTACAACGTGCTGGAGCGG
CCCCGCGGCTGGGCCTTCGTCTACCACGTCTTCATATTTTTGCTGGTCTTCAGCTGTCTGGTGCTGTCTGTGCTGT
CCACTATCCAGGAGCACCAGGAACTTGCCAACGAGTGTCTCCTCATCTTGGAATTCGTGA

**GFP-Kv7.4$^{W242X}$(GFP-linker-Kv7.4$^{W242X}$)**
ATGGTGAGCAAGGGCGAGGAGCTGTTCACCGGGGTGGTGCCCATCCTGGTCGAGCTGGACGGCGACGTAAACGGCC
ACAAGTTCAGCGTGTCCGGCGAGGGCGAGGGCGATGCCACCTACGGCAAGCTGACCCTGAAGTTCATCTGCACCAC
CGGCAAGCTGCCCGTGCCCTGGCCCACCCTCGTGACCACCCTGACCTACGGCGTGCAGTGCTTCAGCCGCTACCCC
GACCACATGAAGCAGCACGACTTCTTCAAGTCCGCCATGCCCGAAGGCTACGTCCAGGAGCGCACCATCTTCTTCA
AGGACGACGGCAACTACAAGACCCGCGCCGAGGTGAAGTTCGAGGGCGACACCCTGGTGAACCGCATCGAGCTGAA
GGGCATCGACTTCAAGGAGGACGGCAACATCCTGGGGCACAAGCTGGAGTACAACTACAACAGCCACAACGTCTAT
ATCATGGCCGACAAGCAGAAGAACGGCATCAAGGTGAACTTCAAGATCCGCCACAACATCGAGGACGGCAGCGTGC
AGCTCGCCGACCACTACCAGCAGAACACCCCCATCGGCGACGGCCCCGTGCTGCTGCCCGACAACCACTACCTGAG
CACCCAGTCCGCCCTGAGCAAAGACCCCAACGAGAAGCGCGATCACATGGTCCTGCTGGAGTTCGTGACCGCCGCC
GGGATCACTCTCGGCATGGACGAGCTGTACAAGTCCGGACTCAGATCTCGAGCTCAAGCTTCGAATTCTGCAGTCG
ACGGTACCGCGGGCCCGGGATCCACCATGGCCGAGGCCCCCCCGCGCCGCCTCGGCCTGGGCCCCCCGCCCGGGGA
CGCCCCCCGCGCGGAGTTGGTGGCGCTCACGGCCGTGCAGAGTGAACAGGGCGAGGCGGGCGGGGGCGGCTCTCCG
CGTCGCCTCGGCCTTCTGGGCAGCCCCCTGCCGCCGGGCGCGCCCCTCCCTGGGCCGGGCTCCGGCTCGGGCTCCG
CCTGCGGCGGCcAGCGCTCCTCCGCCGCGCAGAAGCGCTACCGCCGCCTGCAGAACTGGGTCTACAACGTGCTGGA
GCGGCCCCGCGGGTGGGCCTTCGTCTACCACGTCTTCATATTTTTGCTAGTCTTCAGCTGCCTGGTGCTGTCTGTA
CTGTCCACCATCCAGGAGCACCAGGAACTTGCCAACGAGTGTCTCCTTATCTTGGAATTCGTGATGATTGTGGTCT
TTGGCTTGGAGTATATAATCCGTGTCTGGTCGGCCGGATGCTGTTGTCGCTACAGAGGATGGCAGGGACGCTTTCG
CTTCGCCAGGAAACCCTTCTGTGTCATCGACTTCATCGTGTTCGTGGCCTCGGTGGCAGTGATAGCTGCGGGCACA
CAAGGCAACATCTTTGCTACGTCCGCGTTGCGCAGTATGCGCTTCCTACAGATCCTGCGCATGGTGCGTATGGATC
GCCGCGGTGGCACCTGGAAGCTGTTGGGATCCGTGGTCTATGCGCACAGTAAGGAGCTGATCACCGCCTAG

**GFP-Kv7.4$^{A349fs}$(GFP-linker-Kv7.4$^{A349fs}$)**
ATGGTGAGCAAGGGCGAGGAGCTGTTCACCGGGGTGGTGCCCATCCTGGTCGAGCTGGACGGCGACGTAAACGGCC
ACAAGTTCAGCGTGTCCGGCGAGGGCGAGGGCGATGCCACCTACGGCAAGCTGACCCTGAAGTTCATCTGCACCAC
CGGCAAGCTGCCCGTGCCCTGGCCCACCCTCGTGACCACCCTGACCTACGGCGTGCAGTGCTTCAGCCGCTACCCC
GACCACATGAAGCAGCACGACTTCTTCAAGTCCGCCATGCCCGAAGGCTACGTCCAGGAGCGCACCATCTTCTTCA
AGGACGACGGCAACTACAAGACCCGCGCCGAGGTGAAGTTCGAGGGCGACACCCTGGTGAACCGCATCGAGCTGAA
GGGCATCGACTTCAAGGAGGACGGCAACATCCTGGGGCACAAGCTGGAGTACAACTACAACAGCCACAACGTCTAT
ATCATGGCCGACAAGCAGAAGAACGGCATCAAGGTGAACTTCAAGATCCGCCACAACATCGAGGACGGCAGCGTGC
AGCTCGCCGACCACTACCAGCAGAACACCCCCATCGGCGACGGCCCCGTGCTGCTGCCCGACAACCACTACCTGAG
CACCCAGTCCGCCCTGAGCAAAGACCCCAACGAGAAGCGCGATCACATGGTCCTGCTGGAGTTCGTGACCGCCGCC
GGGATCACTCTCGGCATGGACGAGCTGTACAAGTCCGGACTCAGATCTCGAGCTCAAGCTTCGAATTCTGCAGTCG
ACGGTACCGCGGGCCCGGGATCCACCATGGCCGAGGCCCCCCCGCGCCGCCTCGGCCTGGGTCCCCCGCCCGGGGA
CGCCCCCCGCGCGGAGCTAGTGGCGCTCACGGCCGTGCAGAGCGAACAGGGCGAGGCGGGCGGGGGCGGCTCCCCG
CGCCGCCTCGGCCTCCTGGGCAGCCCCCTGCCGCCGGGCGCGCCCCTCCCTGGGCCGGGCTCCGGCTCGGGCTCCG
CCTGCGGCCAGCGCTCCTCGGCCGCGCACAAGCGCTACCGCCGCCTGCAGAACTGGGTCTACAACGTGCTGGAGCG
GCCCCGCGGCTGGGCCTTCGTCTACCACGTCTTCATATTTTTGCTGGTCTTCAGCTGTCTGGTGCTGTCTGTGCTG
TCCACTATCCAGGAGCACCAGGAACTTGCCAACGAGTGTCTCCTCATCTTGGAATTCGTGATGATCGTGGTTTTCG
GCTTGGAGTACATCGTCCGGGTCTGGTCCGCCGGATGCTGCTGCCGCTACCGAGGATGGCAGGGTCGCTTCCGCTT
TGCCAGAAAGCCCTTCTGTGTCATCGACTTCATCGTGTTCGTGGCCTCGGTGGCCGTCATCGCCGCGGGTACCCAG
GGCAACATCTTCGCCACGTCCGCGCTGCGCAGCATGCGCTTCCTGCAGATCCTGCGCATGGTGCGCATGGACCGCC
GCGGCGGCACCTGGAAGCTGCTGGGCTCAGTGGTCTACGCGCATAGCAAGGAGCTGATCACCGCCTGGTACATCGG
GTTCCTGGTGCTCATCTTCGCCTCCTTCCTGGTCTACCTGGCCGAGAAGGACGCCAACTCCGACTTCTCCTCCTAC
GCCGACTCGCTCTGGTGGGGGACGATTACATTGACAACCATCGGCTATGGTGACAAGACACCGCACACATGGCTGG
GCAGGGTCCTGGCTGCTGGCTTCGCCTTACTGGGCATCTCTTTCTTTGCCCTGCCTGCCGGCATCCTAGGCTCCGG
CTTTGCCCTGAAGGTCCAGGAGCAGCACCGGCAGAAGCACTTCGAGAAGCGGAGGATGCCGGCAGCCAACCTCATC
CAGGCGCCTGTACTCCACCGATATGAGCCGGGCCTACCTGACAGCCACCTGGTACTACTATGA

**GFP-Kv7.4$^{G285S}$(GFP-linker-Kv7.4$^{G285S}$)**

ATGGTGAGCAAGGGCGAGGAGCTGTTCACCGGGGTGGTGCCCATCCTGGTCGAGCTGGACGGCGACGTAAACGGCC
ACAAGTTCAGCGTGTCCGGCGAGGGCGAGGGCGATGCCACCTACGGCAAGCTGACCCTGAAGTTCATCTGCACCAC
CGGCAAGCTGCCCGTGCCCTGGCCCACCCTCGTGACCACCCTGACCTACGGCGTGCAGTGCTTCAGCCGCTACCCC
GACCACATGAAGCAGCACGACTTCTTCAAGTCCGCCATGCCCGAAGGCTACGTCCAGGAGCGCACCATCTTCTTCA
AGGACGACGGCAACTACAAGACCCGCGCCGAGGTGAAGTTCGAGGGCGACACCCTGGTGAACCGCATCGAGCTGAA
GGGCATCGACTTCAAGGAGGACGGCAACATCCTGGGGCACAAGCTGGAGTACAACTACAACAGCCACAACGTCTAT
ATCATGGCCGACAAGCAGAAGAACGGCATCAAGGTGAACTTCAAGATCCGCCACAACATCGAGGACGGCAGCGTGC
AGCTCGCCGACCACTACCAGCAGAACACCCCCATCGGCGACGGCCCCGTGCTGCTGCCCGACAACCACTACCTGAG
CACCCAGTCCGCCCTGAGCAAAGACCCCAACGAGAAGCGCGATCACATGGTCCTGCTGGAGTTCGTGACCGCCGCC
GGGATCACTCTCGGCATGGACGAGCTGTACAAGTCCGGACTCAGATCTCGAGCTCAAGCTTCGAATTCTGCAGTCG
ACGGTACCGCGGGCCCGGGATCCACCATGGCCGAGGCCCCCCGCGCCGCCTCGGCCTGGGTCCCCCGCCCGGGGA
CGCCCCCCGCGCGGAGCTAGTGGCGCTCACGGCCGTGCAGAGCGAACAGGGCGAGGCGGGCGGGGGCGGCTCCCCG
CGCCGCCTCGGCCtCCTGGGCAGCCCCCTGCCGCCGGGCGCGCCCCTCCCTGGGCCGGGCTCCGGCTCGGGCTCCG
CCTGCGGCcAGCGCTCCTCGGCCGCGCACAAGCGCTACCGCCGCCTGCAGAACTGGGTCTACAACGTGCTGGAGCG
GCCCCGCGGCTGGGCCTTCGTCTACCACGTCTTCATATTTTTGCTGGTCTTCAGCTGTCTGGTGCTGTCTGTGCTG
TCCACTATCCAGGAGCACCAGGAACTTGCCAACGAGTGTCTCCTCATCTTGGAATTCGTGATGATCGTGGTTTTCG
GCTTGGAGTACATCGTCCGGGTCTGGTCCGCCGGATGCTGCTGCCGCTACCGAGGATGGCAGGGTCGCTTCCGCTT
TGCCAGAAAGCCCTTCTGTGTCATCGACTTCATCGTGTTCGTGGCCTCGGTGGCCGTCATCGCCGCGGGTACCCAG
GGCAACATCTTCGCCACGTCCGCGCTGCGCAGCATGCGCTTCCTGCAGATCCTGCGCATGGTGCGCATGGACCGCC
GCGGCGGCACCTGGAAGCTGCTGGGCTCAGtGGTCTACGCGCaTAGCAAGGAGCTGATCACCGCCTGGTACATCGG
GTTCCTGGTGCTCATCTTCgCCTCCTTCCTGGTCTACCTGGCCGAGAAGGACGCCAACTCCGACTTCTCCTcctAC
GCCGACTCGCTCTGGTgGGGGACGATTACATTGACAACCATCAGCTATGGTGACAAGACACcGCACACATGGCTGG
GCAGGGTCCTGGCTGCTGGCTTCGCCTTACTGGGCATCTCTTTCTTTGCCCTGCCTGCCGGCATCCTAGGCTCCGG
CTTTGCCCTGAAGGTCCAGGAGCAGCACCGGCAGAAGCACTTCGAGAAGCGGAGGATGCCGGCAGCCAACCTCATC
CAGGCTGCCTGGCGCCTGTACTCCACCGATATGAGCCGGGCCTACCTGACAGCCACCTGGTACTACTATGACAGTA
TCCTCCCATCCTTCAGAGAGCTGGCCCTCTTGTTTGAGCACGTGCAACGGGCCCGCAATGGGGGCCTACGGCCCCT
GGAGGTGCGGCGGGCGCCGGTACCCGACGGAGCACCCTCCCGTTACCCGCCCGTTGCCACCTGCCACCGGCCGGGC
AGCACCTCCTTCTGCCCTGGGGAAAGCAGCCGGATGGGCATCAAAGACCGCATCCGCATGGGCAGCTCCCAGCGGC
GGACGGGTCCTTCCAAGCAGCATCTGGCACCTCCAACAATGCCCACCTCCCCAAGCAGCGAGCAGGTGGGTGAGGC
CACCAGCCCCACCAAGGTGCAAAAGAGCTGGAGCTTCAATGACCGCACCCGCTTCCGGGCATCTCTGAGACTCAAA
CCCCGCACCTCTGCTGAGGATGCCCCCTCAGAGGAAGTAGCAGAGGAGAAGAGCTACCAGTGTGAGCTCACGGTGG
ACGACATCATGCCTGCTGTGAAGACAGTCATCCGCTCCATCAGGATTCTCAAGTTCCTGGTGGCCAAAAGGAAATT
CAAGGAGACACTGCGACCGTACGACGTGAAGGACGTCATTGAGCAGTACTCAGCAGGCCACCTGGACATGCTGGGC
CGgATCAAGAGCCTGCAAACTCGGGTGGACCAAATTGTGGGTCGGGGGCCCGGGGACAGGAAGGCCCGGGAGAAGG
GCGACAAGGGGCCCTCCGACGCGGAGGTGGTGGATGAAATCAGCATGATGGGACGCGTGGTCAAGGTGGAGAAGCA
GGTGCAGTCCATCGAGCACAAGCTGGACCTGCTGTTGGGCTTCTATTCGCGCTGCCTGCGCTCTGGCACCTCGGCC
AGCCTGGGCGCCGTGCAAGTGCCGCTGTTCGACCCCGACATCACCTCCGACTACCACAGCCCTGTGGACCACGAGG
ACATCTCCGTCTCCGCACAGACGCTCAGCATCTCCCGCTCGGTCAGCACCAACATGGACTGA

**GFP-Kv7.4$^{P291L}$ (GFP-linker-Kv7.4$^{P291L}$)**

ATGGTGAGCAAGGGCGAGGAGCTGTTCACCGGGGTGGTGCCCATCCTGGTCGAGCTGGACGGCGACGTAAACGGCC
ACAAGTTCAGCGTGTCCGGCGAGGGCGAGGGCGATGCCACCTACGGCAAGCTGACCCTGAAGTTCATCTGCACCAC
CGGCAAGCTGCCCGTGCCCTGGCCCACCCTCGTGACCACCCTGACCTACGGCGTGCAGTGCTTCAGCCGCTACCCC
GACCACATGAAGCAGCACGACTTCTTCAAGTCCGCCATGCCCGAAGGCTACGTCCAGGAGCGCACCATCTTCTTCA
AGGACGACGGCAACTACAAGACCCGCGCCGAGGTGAAGTTCGAGGGCGACACCCTGGTGAACCGCATCGAGCTGAA
GGGCATCGACTTCAAGGAGGACGGCAACATCCTGGGGCACAAGCTGGAGTACAACTACAACAGCCACAACGTCTAT
ATCATGGCCGACAAGCAGAAGAACGGCATCAAGGTGAACTTCAAGATCCGCCACAACATCGAGGACGGCAGCGTGC
AGCTCGCCGACCACTACCAGCAGAACACCCCCATCGGCGACGGCCCCGTGCTGCTGCCCGACAACCACTACCTGAG
CACCCAGTCCGCCCTGAGCAAAGACCCCAACGAGAAGCGCGATCACATGGTCCTGCTGGAGTTCGTGACCGCCGCC
GGGATCACTCTCGGCATGGACGAGCTGTACAAGTCCGGACTCAGATCTCGAGCTCAAGCTTCGAATTCTGCAGTCG
ACGGTACCGCGGGCCCGGGATCCACCATGGCCGAGGCCCCCCGCGCCGCCTCGGCCTGGGTCCCCCGCCCGGGGA
CGCCCCCCGCGCGGAGCTAGTGGCGCTCACGGCCGTGCAGAGCGAACAGGGCGAGGCGGGCGGGGGCGGCTCCCCG
CGCCGCCTCGGCCtCCTGGGCAGCCCCCTGCCGCCGGGCGCGCCCCTCCCTGGGCCGGGCTCCGGCTCGGGCTCCG
CCTGCGGCcAGCGCTCCTCGGCCGCGCACAAGCGCTACCGCCGCCTGCAGAACTGGGTCTACAACGTGCTGGAGCG

GCCCCGCGGCTGGGCCTTCGTCTACCACGTCTTCATATTTTTGCTGGTCTTCAGCTGTCTGGTGCTGTCTGTGCTG
TCCACTATCCAGGAGCACCAGGAACTTGCCAACGAGTGTCTCCTCATCTTGGAATTCGTGATGATCGTGGTTTTCG
GCTTGGAGTACATCGTCCGGGTCTGGTCCGCCGGATGCTGCTGCCGCTACCGAGGATGGCAGGGTCGCTTCCGCTT
TGCCAGAAAGCCCTTCTGTGTCATCGACTTCATCGTGTTCGTGGCCTCGGTGGCCGTCATCGCCGCGGGTACCCAG
GGCAACATCTTCGCCACGTCCGCGCTGCGCAGCATGCGCTTCCTGCAGATCCTGCGCATGGTGCGCATGGACCGCC
GCGGCGGCACCTGGAAGCTGCTGGGCTCAGtGGTCTACGCGCaTAGCAAGGAGCTGATCACCGCCTGGTACATCGG
GTTCCTGGTGCTCATCTTCgCCTCCTTCCTGGTCTACCTGGCCGAGAAGGACGCCAACTCCGACTTCTCCTcctAC
GCCGACTCGCTCTGGTgGGGGACGATTACATTGACAACCATCgGCTATGGTGACAAGACACTGCACACATGGCTGG
GCAGGGTCCTGGCTGCTGGCTTCGCCTTACTGGGCATCTCTTTCTTTGCCCTGCCTGCCGGCATCCTAGGCTCCGG
CTTTGCCCTGAAGGTCCAGGAGCAGCACCGGCAGAAGCACTTCGAGAAGCGGAGGATGCCGGCAGCCAACCTCATC
CAGGCTGCCTGGCGCCTGTACTCCACCGATATGAGCCGGGCCTACCTGACAGCCACCTGGTACTACTATGACAGTA
TCCTCCCATCCTTCAGAGAGCTGGCCCTCTTGTTTGAGCACGTGCAACGGGCCCGCAATGGGGGCCTACGGCCCCT
GGAGGTGCGGCGGGCGCCGGTACCCGACGGAGCACCCTCCCGTTACCCGCCCGTTGCCACCTGCCACCGGCCGGGC
AGCACCTCCTTCTGCCCTGGGGAAAGCAGCCGGATGGGCATCAAAGACCGCATCCGCATGGGCAGCTCCCAGCGGC
GGACGGGTCCTTCCAAGCAGCATCTGGCACCTCCAACAATGCCCACCTCCCCAAGCAGCGAGCAGGTGGGTGAGGC
CACCAGCCCCACCAAGGTGCAAAAGAGCTGGAGCTTCAATGACCGCACCCGCTTCCGGGCATCTCTGAGACTCAAA
CCCCGCACCTCTGCTGAGGATGCCCCCTCAGAGGAAGTAGCAGAGGAGAAGAGCTACCAGTGTGAGCTCACGGTGG
ACGACATCATGCCTGCTGTGAAGACAGTCATCCGCTCCATCAGGATTCTCAAGTTCCTGGTGGCCAAAAGGAAATT
CAAGGAGACACTGCGACCGTACGACGTGAAGGACGTCATTGAGCAGTACTCAGCAGGCCACCTGGACATGCTGGGC
CGgATCAAGAGCCTGCAAACTCGGGTGGACCAAATTGTGGGTCGGGGGCCCGGGGACAGGAAGGCCCGGGAGAAGG
GCGACAAGGGGCCCTCCGACGCGGAGGTGGTGGATGAAATCAGCATGATGGGACGCGTGGTCAAGGTGGAGAAGCA
GGTGCAGTCCATCGAGCACAAGCTGGACCTGCTGTTGGGCTTCTATTCGCGCTGCCTGCGCTCTGGCACCTCGGCC
AGCCTGGGCGCCGTGCAAGTGCCGCTGTTCGACCCCGACATCACCTCCGACTACCACAGCCCTGTGGACCACGAGG
ACATCTCCGTCTCCGCACAGACGCTCAGCATCTCCCGCTCGGTCAGCACCAACATGGACTGA

**Kv7.4$^{WT}$-P2A-Kv7.4$^{Q71fs}$ (Kv7.4$^{WT}$-P2A-Kv7.4$^{Q71fs}$)**

ATGGCCGAGGCCCCCCCGCGCCGCCTCGGCCTGGGTCCCCCGCCCGGGGACGCCCCCCGCGCGGAGCTAGTGGCGC
TCACGGCCGTGCAGAGCGAACAGGGCGAGGCGGGCGGGGGCGGCTCCCCGCGCCGCCTCGGCCTCCTGGGCAGCCC
CCTGCCGCCGGGCGCGCCCCTCCCTGGGCCGGGCTCCGGCTCGGGCTCCGCCTGCGGCCAGCGCTCCTCGGCCGCG
CACAAGCGCTACCGCCGCCTGCAGAACTGGGTCTACAACGTGCTGGAGCGGCCCCGCGGCTGGGCCTTCGTCTACC
ACGTCTTCATATTTTTGCTGGTCTTCAGCTGTCTGGTGCTGTCTGTGCTGTCCACTATCCAGGAGCACCAGGAACT
TGCCAACGAGTGTCTCCTCATCTTGGAATTCGTGATGATCGTGGTTTTCGGCTTGGAGTACATCGTCCGGGTCTGG
TCCGCCGGATGCTGCTGCCGCTACCGAGGATGGCAGGGTCGCTTCCGCTTTGCCAGAAAGCCCTTCTGTGTCATCG
ACTTCATCGTGTTCGTGGCCTCGGTGGCCGTCATCGCCGCGGGTACCCAGGGCAACATCTTCGCCACGTCCGCGCT
GCGCAGCATGCGCTTCCTGCAGATCCTGCGCATGGTGCGCATGGACCGCCGCGGCGGCACCTGGAAGCTGCTGGGC
TCAGTGGTCTACGCGCATAGCAAGGAGCTGATCACCGCCTGGTACATCGGGTTCCTGGTGCTCATCTTCGCCTCCT
TCCTGGTCTACCTGGCCGAGAAGGACGCCAACTCCGACTTCTCCTCCTACGCCGACTCGCTCTGGTGGGGGACGAT
TACATTGACAACCATCGGCTATGGTGACAAGACACCGCACACATGGCTGGGCAGGGTCCTGGCTGCTGGCTTCGCC
TTACTGGGCATCTCTTTCTTTGCCCTGCCTGCCGGCATCCTAGGCTCCGGCTTTGCCCTGAAGGTCCAGGAGCAGC
ACCGGCAGAAGCACTTCGAGAAGCGGAGGATGCCGGCAGCCAACCTCATCCAGGCTGCCTGGCGCCTGTACTCCAC
CGATATGAGCCGGGCCTACCTGACAGCCACCTGGTACTACTATGACAGTATCCTCCCATCCTTCAGAGAGCTGGCC
CTCTTGTTTGAGCACGTGCAACGGGCCCGCAATGGGGGCCTACGGCCCCTGGAGGTGCGGCGGGCGCCGGTACCCG
ACGGAGCACCCTCCCGTTACCCGCCCGTTGCCACCTGCCACCGGCCGGGCAGCACCTCCTTCTGCCCTGGGGAAAG
CAGCCGGATGGGCATCAAAGACCGCATCCGCATGGGCAGCTCCCAGCGGCGGACGGGTCCTTCCAAGCAGCATCTG
GCACCTCCAACAATGCCCACCTCCCCAAGCAGCGAGCAGGTGGGTGAGGCCACCAGCCCCACCAAGGTGCAAAAGA
GCTGGAGCTTCAATGACCGCACCCGCTTCCGGGCATCTCTGAGACTCAAACCCCGCACCTCTGCTGAGGATGCCCC
CTCAGAGGAAGTAGCAGAGGAGAAGAGCTACCAGTGTGAGCTCACGGTGGACGACATCATGCCTGCTGTGAAGACA
GTCATCCGCTCCATCAGGATTCTCAAGTTCCTGGTGGCCAAAAGGAAATTCAAGGAGACACTGCGACCGTACGACG
TGAAGGACGTCATTGAGCAGTACTCAGCAGGCCACCTGGACATGCTGGGCCGGATCAAGAGCCTGCAAACTCGGGT
GGACCAAATTGTGGGTCGGGGGCCCGGGGACAGGAAGGCCCGGGAGAAGGGCGACAAGGGGCCCTCCGACGCGGAG
GTGGTGGATGAAATCAGCATGATGGGACGCGTGGTCAAGGTGGAGAAGCAGGTGCAGTCCATCGAGCACAAGCTGG
ACCTGCTGTTGGGCTTCTATTCGCGCTGCCTGCGCTCTGGCACCTCGGCCAGCCTGGGCGCCGTGCAAGTGCCGCT
GTTCGACCCCGACATCACCTCCGACTACCACAGCCCTGTGGACCACGAGGACATCTCCGTCTCCGCACAGACGCTC
AGCATCTCCCGCTCGGTCAGCACCAACATGGACGGAAGCGGAGCTACTAACTTCAGCCTGCTGAAGCAGGCTGGAG
ACGTCGAGGAGAACCCTGGACCTATGGCCGAGGCCCCCCCGCGCCGCCTCGGCCTGGGTCCCCCGCCCGGGGACGC
CCCCCGCGCGGAGCTAGTGGCGCTCACGGCCGTGCAGAGCGAACAGGGCGAGGCGGGCGGGGGCGGCTCCCCGCGC

Disease Models & Mechanisms • Supplementary information

CGCCTCGGCCTCCTGGGCAGCCCCCTGCCGCCGGGCGCGCCCCTCCCTGGGCCGGGCTCCGGCTCGGGCTCCGCCT
GCGGCAGCGCTCCTCGGCCGCGCACAAGCGCTACCGCCGCCTGCAGAACTGGGTCTACAACGTGCTGGAGCGGCCC
CGCGGCTGGGCCTTCGTCTACCACGTCTTCATATTTTTGCTGGTCTTCAGCTGTCTGGTGCTGTCTGTGCTGTCCA
CTATCCAGGAGCACCAGGAACTTGCCAACGAGTGTCTCCTCATCTTGGAATTCGTGA

**Kv7.4$^{WT}$-P2A-Kv7.4$^{W242X}$ (Kv7.4$^{WT}$-P2A-Kv7.4$^{W242X}$)**

ATGGCCGAGGCCCCCCCGCGCCGCCTCGGCCTGGGTCCCCCGCCCGGGGACGCCCCCCGCGCGGAGCTAGTGGCGC
TCACGGCCGTGCAGAGCGAACAGGGCGAGGCGGGCGGGGGCGGCTCCCCGCGCCGCCTCGGCCTCCTGGGCAGCCC
CCTGCCGCCGGGCGCGCCCCTCCCTGGGCCGGGCTCCGGCTCGGGCTCCGCCTGCGGCCAGCGCTCCTCGGCCGCG
CACAAGCGCTACCGCCGCCTGCAGAACTGGGTCTACAACGTGCTGGAGCGGCCCCGCGGCTGGGCCTTCGTCTACC
ACGTCTTCATATTTTTGCTGGTCTTCAGCTGTCTGGTGCTGTCTGTGCTGTCCACTATCCAGGAGCACCAGGAACT
TGCCAACGAGTGTCTCCTCATCTTGGAATTCGTGATGATCGTGGTTTTCGGCTTGGAGTACATCGTCCGGGTCTGG
TCCGCCGGATGCTGCTGCCGCTACCGAGGATGGCAGGGTCGCTTCCGCTTTGCCAGAAAGCCCTTCTGTGTCATCG
ACTTCATCGTGTTCGTGGCCTCGGTGGCCGTCATCGCCGCGGGTACCCAGGGCAACATCTTCGCCACGTCCGCGCT
GCGCAGCATGCGCTTCCTGCAGATCCTGCGCATGGTGCGCATGGACCGCCGCGGCGGCACCTGGAAGCTGCTGGGC
TCAGTGGTCTACGCGCATAGCAAGGAGCTGATCACCGCCTGGTACATCGGGTTCCTGGTGCTCATCTTCGCCTCCT
TCCTGGTCTACCTGGCCGAGAAGGACGCCAACTCCGACTTCTCCTCCTACGCCGACTCGCTCTGGTGGGGGACGAT
TACATTGACAACCATCGGCTATGGTGACAAGACACCGCACACATGGCTGGGCAGGGTCCTGGCTGCTGGCTTCGCC
TTACTGGGCATCTCTTTCTTTGCCCTGCCTGCCGGCATCCTAGGCTCCGGCTTTGCCCTGAAGGTCCAGGAGCAGC
ACCGGCAGAAGCACTTCGAGAAGCGGAGGATGCCGGCAGCCAACCTCATCCAGGCTGCCTGGCGCCTGTACTCCAC
CGATATGAGCCGGGCCTACCTGACAGCCACCTGGTACTACTATGACAGTATCCTCCCATCCTTCAGAGAGCTGGCC
CTCTTGTTTGAGCACGTGCAACGGGCCCGCAATGGGGGCCTACGGCCCCTGGAGGTGCGGCGGGCGCCGGTACCCG
ACGGAGCACCCTCCCGTTACCCGCCCGTTGCCACCTGCCACCGGCCGGGCAGCACCTCCTTCTGCCCTGGGGAAAG
CAGCCGGATGGGCATCAAAGACCGCATCCGCATGGGCAGCTCCCAGCGGCGGACGGGTCCTTCCAAGCAGCATCTG
GCACCTCCAACAATGCCCACCTCCCCAAGCAGCGAGCAGGTGGGTGAGGCCACCAGCCCCACCAAGGTGCAAAAGA
GCTGGAGCTTCAATGACCGCACCCGCTTCCGGGCATCTCTGAGACTCAAACCCCGCACCTCTGCTGAGGATGCCCC
CTCAGAGGAAGTAGCAGAGGAGAAGAGCTACCAGTGTGAGCTCACGGTGGACGACATCATGCCTGCTGTGAAGACA
GTCATCCGCTCCATCAGGATTCTCAAGTTCCTGGTGGCCAAAAGGAAATTCAAGGAGACACTGCGACCGTACGACG
TGAAGGACGTCATTGAGCAGTACTCAGCAGGCCACCTGGACATGCTGGGCCGGATCAAGAGCCTGCAAACTCGGGT
GGACCAAATTGTGGGTCGGGGGCCCGGGGACAGGAAGGCCCGGGAGAAGGGCGACAAGGGGCCCTCCGACGCGGAG
GTGGTGGATGAAATCAGCATGATGGGACGCGTGGTCAAGGTGGAGAAGCAGGTGCAGTCCATCGAGCACAAGCTGG
ACCTGCTGTTGGGCTTCTATTCGCGCTGCCTGCGCTCTGGCACCTCGGCCAGCCTGGGCGCCGTGCAAGTGCCGCT
GTTCGACCCCGACATCACCTCCGACTACCACAGCCCTGTGGACCACGAGGACATCTCCGTCTCCGCACAGACGCTC
AGCATCTCCCGCTCGGTCAGCACCAACATGGACGGAAGCGGAGCTACTAACTTCAGCCTGCTGAAGCAGGCTGGAG
ACGTCGAGGAGAACCCTGGACCTATGGCCGAGGCCCCCCCGCGCCGCCTCGGCCTGGGCCCCCCGCCCGGGGACGC
CCCCCGCGCGGAGTTGGTGGCGCTCACGGCCGTGCAGAGTGAACAGGGCGAGGCGGGCGGGGGCGGCTCTCCGCGT
CGCCTCGGCCTTCTGGGCAGCCCCCTGCCGCCGGGCGCGCCCCTCCCTGGGCCGGGCTCCGGCTCGGGCTCCGCCT
GCGGCGGCcAGCGCTCCTCCGCCGCGCAGAAGCGCTACCGCCGCCTGCAGAACTGGGTCTACAACGTGCTGGAGCG
GCCCCGCGGGTGGGCCTTCGTCTACCACGTCTTCATATTTTTGCTAGTCTTCAGCTGCCTGGTGCTGTCTGTACTG
TCCACCATCCAGGAGCACCAGGAACTTGCCAACGAGTGTCTCCTTATCTTGGAATTCGTGATGATTGTGGTCTTTG
GCTTGGAGTATATAATCCGTGTCTGGTCGGCCGGATGCTGTTGTCGCTACAGAGGATGGCAGGGACGCTTTCGCTT
CGCCAGGAAACCCTTCTGTGTCATCGACTTCATCGTGTTCGTGGCCTCGGTGGCAGTGATAGCTGCGGGCACACAA
GGCAACATCTTTGCTACGTCCGCGTTGCGCAGTATGCGCTTCCTACAGATCCTGCGCATGGTGCGTATGGATCGCC
GCGGTGGCACCTGGAAGCTGTTGGGATCCGTGGTCTATGCGCACAGTAAGGAGCTGATCACCGCCTAG

**Kv7.4$^{WT}$-P2A-Kv7.4$^{A349fs}$ (Kv7.4$^{WT}$-P2A-Kv7.4$^{A349fs}$)**

ATGGCCGAGGCCCCCCCGCGCCGCCTCGGCCTGGGTCCCCCGCCCGGGGACGCCCCCCGCGCGGAGCTAGTGGCGC
TCACGGCCGTGCAGAGCGAACAGGGCGAGGCGGGCGGGGGCGGCTCCCCGCGCCGCCTCGGCCTCCTGGGCAGCCC
CCTGCCGCCGGGCGCGCCCCTCCCTGGGCCGGGCTCCGGCTCGGGCTCCGCCTGCGGCCAGCGCTCCTCGGCCGCG
CACAAGCGCTACCGCCGCCTGCAGAACTGGGTCTACAACGTGCTGGAGCGGCCCCGCGGCTGGGCCTTCGTCTACC
ACGTCTTCATATTTTTGCTGGTCTTCAGCTGTCTGGTGCTGTCTGTGCTGTCCACTATCCAGGAGCACCAGGAACT
TGCCAACGAGTGTCTCCTCATCTTGGAATTCGTGATGATCGTGGTTTTCGGCTTGGAGTACATCGTCCGGGTCTGG
TCCGCCGGATGCTGCTGCCGCTACCGAGGATGGCAGGGTCGCTTCCGCTTTGCCAGAAAGCCCTTCTGTGTCATCG
ACTTCATCGTGTTCGTGGCCTCGGTGGCCGTCATCGCCGCGGGTACCCAGGGCAACATCTTCGCCACGTCCGCGCT
GCGCAGCATGCGCTTCCTGCAGATCCTGCGCATGGTGCGCATGGACCGCCGCGGCGGCACCTGGAAGCTGCTGGGC
TCAGTGGTCTACGCGCATAGCAAGGAGCTGATCACCGCCTGGTACATCGGGTTCCTGGTGCTCATCTTCGCCTCCT

TCCTGGTCTACCTGGCCGAGAAGGACGCCAACTCCGACTTCTCCTCCTACGCCGACTCGCTCTGGTGGGGACGAT
TACATTGACAACCATCGGCTATGGTGACAAGACACCGCACACATGGCTGGGCAGGGTCCTGGCTGCTGGCTTCGCC
TTACTGGGCATCTCTTTCTTTGCCCTGCCTGCCGGCATCCTAGGCTCCGGCTTTGCCCTGAAGGTCCAGGAGCAGC
ACCGGCAGAAGCACTTCGAGAAGCGGAGGATGCCGGCAGCCAACCTCATCCAGGCTGCCTGGCGCCTGTACTCCAC
CGATATGAGCCGGGCCTACCTGACAGCCACCTGGTACTACTATGACAGTATCCTCCCATCCTTCAGAGAGCTGGCC
CTCTTGTTTGAGCACGTGCAACGGGCCCGCAATGGGGGCCTACGGCCCCTGGAGGTGCGGCGGGCGCCGGTACCCG
ACGGAGCACCCTCCCGTTACCCGCCCGTTGCCACCTGCCACCGGCCGGGCAGCACCTCCTTCTGCCCTGGGGAAAG
CAGCCGGATGGGCATCAAAGACCGCATCCGCATGGGCAGCTCCCAGCGGCGGACGGGTCCTTCCAAGCAGCATCTG
GCACCTCCAACAATGCCCACCTCCCCAAGCAGCGAGCAGGTGGGTGAGGCCACCAGCCCCACCAAGGTGCAAAAGA
GCTGGAGCTTCAATGACCGCACCCGCTTCCGGGCATCTCTGAGACTCAAACCCCGCACCTCTGCTGAGGATGCCCC
CTCAGAGGAAGTAGCAGAGGAGAAGAGCTACCAGTGTGAGCTCACGGTGGACGACATCATGCCTGCTGTGAAGACA
GTCATCCGCTCCATCAGGATTCTCAAGTTCCTGGTGGCCAAAAGGAAATTCAAGGAGACACTGCGACCGTACGACG
TGAAGGACGTCATTGAGCAGTACTCAGCAGGCCACCTGGACATGCTGGGCCGGATCAAGAGCCTGCAAACTCGGGT
GGACCAAATTGTGGGTCGGGGGCCCGGGGACAGGAAGGCCCGGGAGAAGGGCGACAAGGGGCCCTCCGACGCGGAG
GTGGTGGATGAAATCAGCATGATGGGACGCGTGGTCAAGGTGGAGAAGCAGGTGCAGTCCATCGAGCACAAGCTGG
ACCTGCTGTTGGGCTTCTATTCGCGCTGCCTGCGCTCTGGCACCTCGGCCAGCCTGGGCGCCGTGCAAGTGCCGCT
GTTCGACCCCGACATCACCTCCGACTACCACAGCCCTGTGGACCACGAGGACATCTCCGTCTCCGCACAGACGCTC
AGCATCTCCCGCTCGGTCAGCACCAACATGGACGGAAGCGGAGCTACTAACTTCAGCCTGCTGAAGCAGGCTGGAG
ACGTCGAGGAGAACCCTGGACCTATGGCCGAGGCCCCCCGCGCCGCCTCGGCCTGGGTCCCCCGCCCGGGGACGC
CCCCCGCGCGGAGCTAGTGGCGCTCACGGCCGTGCAGAGCGAACAGGGCGAGGCGGGCGGGGGCGGCTCCCCGCGC
CGCCTCGGCCTCCTGGGCAGCCCCCTGCCGCCGGGCGCGCCCCTCCCTGGGCCGGGCTCCGGCTCGGGCTCCGCCT
GCGGCCAGCGCTCCTCGGCCGCGCACAAGCGCTACCGCCGCCTGCAGAACTGGGTCTACAACGTGCTGGAGCGGCC
CCGCGGCTGGGCCTTCGTCTACCACGTCTTCATATTTTTGCTGGTCTTCAGCTGTCTGGTGCTGTCTGTGCTGTCC
ACTATCCAGGAGCACCAGGAACTTGCCAACGAGTGTCTCCTCATCTTGGAATTCGTGATGATCGTGGTTTTCGGCT
TGGAGTACATCGTCCGGGTCTGGTCCGCCGGATGCTGCTGCCGCTACCGAGGATGGCAGGGTCGCTTCCGCTTTGC
CAGAAAGCCCTTCTGTGTCATCGACTTCATCGTGTTCGTGGCCTCGGTGGCCGTCATCGCCGCGGGTACCCAGGGC
AACATCTTCGCCACGTCCGCGCTGCGCAGCATGCGCTTCCTGCAGATCCTGCGCATGGTGCGCATGGACCGCCGCG
GCGGCACCTGGAAGCTGCTGGGCTCAGTGGTCTACGCGCATAGCAAGGAGCTGATCACCGCCTGGTACATCGGGTT
CCTGGTGCTCATCTTCGCCTCCTTCCTGGTCTACCTGGCCGAGAAGGACGCCAACTCCGACTTCTCCTCCTACGCC
GACTCGCTCTGGTGGGGACGATTACATTGACAACCATCGGCTATGGTGACAAGACACCGCACACATGGCTGGGCA
GGGTCCTGGCTGCTGGCTTCGCCTTACTGGGCATCTCTTTCTTTGCCCTGCCTGCCGGCATCCTAGGCTCCGGCTT
TGCCCTGAAGGTCCAGGAGCAGCACCGGCAGAAGCACTTCGAGAAGCGGAGGATGCCGGCAGCCAACCTCATCCAG
GCGCCTGTACTCCACCGATATGAGCCGGGCCTACCTGACAGCCACCTGGTACTACTATGA

**Kv7.1**$^{WT}$ (human wild-type)
ATGGCCGCGGCCTCCTCCCCGCCCAGGGCCGAGAGGAAGCGCTGGGGTTGGGGCCGCCTGCCAGGCGCCCGGCGGG
GCAGCGCGGGCCTGGCCAAGAAGTGCCCCTTCTCGCTGGAGCTGGCGGAGGGCGGCCCGGCGGGCGGCGCGCTCTA
CGCGCCCATCGCGCCCGGCGCCCCAGGTCCCGCGCCCCCTGCGTCCCCGGCCGCGCCCGCCGCGCCCCCAGTTGCC
TCCGACCTTGGCCCGCGGCCGCCGGTGAGCCTAGACCCGCGCGTCTCCATCTACAGCACGCGCCGCCCGGTGTTGG
CGCGCACCCACGTCCAGGGCCGCGTCTACAACTTCCTCGAGCGTCCCACCGGCTGGAAATGCTTCGTTTACCACTT
CGCCGTCTTCCTCATCGTCCTGGTCTGCCTCATCTTCAGCGTGCTGTCCACCATCGAGCAGTATGCCGCCCTGGCC
ACGGGGACTCTCTTCTGGATGGAGATCGTGCTGGTGGTGTTCTTCGGGACGGAGTACGTGGTCCGCCTCTGGTCCG
CCGGCTGCCGCAGCAAGTACGTGGGCCTCTGGGGGCGGCTGCGCTTTGCCCGGAAGCCCATTTCCATCATCGACCT
CATCGTGGTCGTGGCCTCCATGGTGGTCCTCTGCGTGGGCTCCAAGGGGCAGGTGTTTGCCACGTCGGCCATCAGG
GGCATCCGCTTCCTGCAGATCCTGAGGATGCTACACGTCGACCGCCAGGGAGGCACCTGGAGGCTCCTGGGCTCCG
TGGTCTTCATCCACCGCCAGGAGCTGATAACCACCCTGTACATCGGCTTCCTGGGCCTCATCTTCTCCTCGTACTT
TGTGTACCTGGCTGAGAAGGACGCGGTGAACGAGTCAGGCCGCGTGGAGTTCGGCAGCTACGCAGATGCGCTGTGG
TGGGGGGTGGTCACAGTCACCACCATCGGCTATGGGGACAAGGTGCCCCAGACGTGGGTCGGGAAGACCATCGCCT
CCTGCTTCTCTGTCTTTGCCATCTCCTTCTTTGCGCTCCCAGCGGGGATTCTTGGCTCGGGGTTTGCCCTGAAGGT
GCAGCAGAAGCAGAGGCAGAAGCACTTCAACCGGCAGATCCCGGCGGCAGCCTCACTCATTCAGACCGCATGGAGG
TGCTATGCTGCCGAGAACCCCGACTCCTCCACCTGGAAGATCTACATCCGGAAGGCCCCCCGGAGCCACACTCTGC
TGTCACCCAGCCCCAAACCCAAGAAGTCTGTGGTGGTAAAGAAAAAAAAGTTCAAGCTGGACAAAGACAATGGGGT
GACTCCTGGAGAGAAGATGCTCACAGTCCCCCATATCACGTGCGACCCCCCAGAAGAGCGGCGGCTGGACCACTTC
TCTGTCGACGGCTATGACAGTTCTGTAAGGAAGAGCCCAACACTGCTGGAAGTGAGCATGCCCCATTTCATGAGAA
CCAACAGCTTCGCCGAGGACCTGGACCTGGAAGGGGAGACTCTGCTGACACCCATCACCCACATCTCACAGCTGCG
GGAACACCATCGGGCCACCATTAAGGTCATTCGACGCATGCAGTACTTTGTGGCCAAGAAGAAATTCCAGCAAGCG

Disease Models & Mechanisms • Supplementary information

CGGAAGCCTTACGATGTGCGGGACGTCATTGAGCAGTACTCGCAGGGCCACCTCAACCTCATGGTGCGCATCAAGG
AGCTGCAGAGGAGGCTGGACCAGTCCATTGGGAAGCCCTCACTGTTCATCTCCGTCTCAGAAAAGAGCAAGGATCG
CGGCAGCAACACGATCGGCGCCCGCCTGAACCGAGTAGAAGACAAGGTGACGCAGCTGGACCAGAGGCTGGCACTC
ATCACCGACATGCTTCACCAGCTGCTCTCCTTGCACGGTGGCAGCACCCCCGGCAGCGGCGGCCCCCCCAGAGAGG
GCGGGGCCCACATCACCCAGCCCTGCGGCAGTGGCGGCTCCGTCGACCCTGAGCTCTTCCTGCCCAGCAACACCCT
GCCCACCTACGAGCAGCTGACCGTGCCCAGGAGGGGCCCCGATGAGGGGTCCTGA

**Kv7.1**$^{E261X}$ (c.781G>T)
ATGGCCGCGGCCTCCTCCCCGCCCAGGGCCGAGAGGAAGCGCTGGGGTTGGGGCCGCCTGCCAGGCGCCCGGCGGG
GCAGCGCGGGCCTGGCCAAGAAGTGCCCCTTCTCGCTGGAGCTGGCGGAGGGCGGCCCGGCGGGCGGCGCGCTCTA
CGCGCCCATCGCGCCCGGCGCCCCAGGTCCCGCGCCCCCTGCGTCCCCGGCCGCGCCCGCCGCGCCCCCAGTTGCC
TCCGACCTTGGCCCGCGGCCGCCGGTGAGCCTAGACCCGCGCGTCTCCATCTACAGCACGCGCCGCCCGGTGTTGG
CGCGCACCCACGTCCAGGGCCGCGTCTACAACTTCCTCGAGCGTCCCACCGGCTGGAAATGCTTCGTTTACCACTT
CGCCGTCTTCCTCATCGTCCTGGTCTGCCTCATCTTCAGCGTGCTGTCCACCATCGAGCAGTATGCCGCCCTGGCC
ACGGGGACTCTCTTCTGGATGGAGATCGTGCTGGTGGTGTTCTTCGGGACGGAGTACGTGGTCCGCCTCTGGTCCG
CCGGCTGCCGCAGCAAGTACGTGGGCCTCTGGGGGCGGCTGCGCTTTGCCCGGAAGCCCATTTCCATCATCGACCT
CATCGTGGTCGTGGCCTCCATGGTGGTCCTCTGCGTGGGCTCCAAGGGGCAGGTGTTTGCCACGTCGGCCATCAGG
GGCATCCGCTTCCTGCAGATCCTGAGGATGCTACACGTCGACCGCCAGGGAGGCACCTGGAGGCTCCTGGGCTCCG
TGGTCTTCATCCACCGCCAGTAG

**Kv7.1**$^{W305X}$ (c.914G>A)
ATGGCCGCGGCCTCCTCCCCGCCCAGGGCCGAGAGGAAGCGCTGGGGTTGGGGCCGCCTGCCAGGCGCCCGGCGGG
GCAGCGCGGGCCTGGCCAAGAAGTGCCCCTTCTCGCTGGAGCTGGCGGAGGGCGGCCCGGCGGGCGGCGCGCTCTA
CGCGCCCATCGCGCCCGGCGCCCCAGGTCCCGCGCCCCCTGCGTCCCCGGCCGCGCCCGCCGCGCCCCCAGTTGCC
TCCGACCTTGGCCCGCGGCCGCCGGTGAGCCTAGACCCGCGCGTCTCCATCTACAGCACGCGCCGCCCGGTGTTGG
CGCGCACCCACGTCCAGGGCCGCGTCTACAACTTCCTCGAGCGTCCCACCGGCTGGAAATGCTTCGTTTACCACTT
CGCCGTCTTCCTCATCGTCCTGGTCTGCCTCATCTTCAGCGTGCTGTCCACCATCGAGCAGTATGCCGCCCTGGCC
ACGGGGACTCTCTTCTGGATGGAGATCGTGCTGGTGGTGTTCTTCGGGACGGAGTACGTGGTCCGCCTCTGGTCCG
CCGGCTGCCGCAGCAAGTACGTGGGCCTCTGGGGGCGGCTGCGCTTTGCCCGGAAGCCCATTTCCATCATCGACCT
CATCGTGGTCGTGGCCTCCATGGTGGTCCTCTGCGTGGGCTCCAAGGGGCAGGTGTTTGCCACGTCGGCCATCAGG
GGCATCCGCTTCCTGCAGATCCTGAGGATGCTACACGTCGACCGCCAGGGAGGCACCTGGAGGCTCCTGGGCTCCG
TGGTCTTCATCCACCGCCAGGAGCTGATAACCACCCTGTACATCGGCTTCCTGGGCCTCATCTTCTCCTCGTACTT
TGTGTACCTGGCTGAGAAGGACGCGGTGAACGAGTCAGGCCGCGTGGAGTTCGGCAGCTACGCAGATGCGCTGTGG
TAG

**Kv7.1**$^{Q530X}$ (c.1588C>T)
ATGGCCGCGGCCTCCTCCCCGCCCAGGGCCGAGAGGAAGCGCTGGGGTTGGGGCCGCCTGCCAGGCGCCCGGCGGG
GCAGCGCGGGCCTGGCCAAGAAGTGCCCCTTCTCGCTGGAGCTGGCGGAGGGCGGCCCGGCGGGCGGCGCGCTCTA
CGCGCCCATCGCGCCCGGCGCCCCAGGTCCCGCGCCCCCTGCGTCCCCGGCCGCGCCCGCCGCGCCCCCAGTTGCC
TCCGACCTTGGCCCGCGGCCGCCGGTGAGCCTAGACCCGCGCGTCTCCATCTACAGCACGCGCCGCCCGGTGTTGG
CGCGCACCCACGTCCAGGGCCGCGTCTACAACTTCCTCGAGCGTCCCACCGGCTGGAAATGCTTCGTTTACCACTT
CGCCGTCTTCCTCATCGTCCTGGTCTGCCTCATCTTCAGCGTGCTGTCCACCATCGAGCAGTATGCCGCCCTGGCC
ACGGGGACTCTCTTCTGGATGGAGATCGTGCTGGTGGTGTTCTTCGGGACGGAGTACGTGGTCCGCCTCTGGTCCG
CCGGCTGCCGCAGCAAGTACGTGGGCCTCTGGGGGCGGCTGCGCTTTGCCCGGAAGCCCATTTCCATCATCGACCT
CATCGTGGTCGTGGCCTCCATGGTGGTCCTCTGCGTGGGCTCCAAGGGGCAGGTGTTTGCCACGTCGGCCATCAGG
GGCATCCGCTTCCTGCAGATCCTGAGGATGCTACACGTCGACCGCCAGGGAGGCACCTGGAGGCTCCTGGGCTCCG
TGGTCTTCATCCACCGCCAGGAGCTGATAACCACCCTGTACATCGGCTTCCTGGGCCTCATCTTCTCCTCGTACTT
TGTGTACCTGGCTGAGAAGGACGCGGTGAACGAGTCAGGCCGCGTGGAGTTCGGCAGCTACGCAGATGCGCTGTGG
TGGGGGGTGGTCACAGTCACCACCATCGGCTATGGGGACAAGGTGCCCCAGACGTGGGTCGGGAAGACCATCGCCT
CCTGCTTCTCTGTCTTTGCCATCTCCTTCTTTGCGCTCCCAGCGGGGATTCTTGGCTCGGGGTTTGCCCTGAAGGT
GCAGCAGAAGCAGAGGCAGAAGCACTTCAACCGGCAGATCCCGGCGGCAGCCTCACTCATTCAGACCGCATGGAGG
TGCTATGCTGCCGAGAACCCCGACTCCTCCACCTGGAAGATCTACATCCGGAAGGCCCCCCGGAGCCACACTCTGC
TGTCACCCAGCCCCAAACCCAAGAAGTCTGTGGTGGTAAAGAAAAAAAAGTTCAAGCTGGACAAAGACAATGGGGT
GACTCCTGGAGAGAAGATGCTCACAGTCCCCCATATCACGTGCGACCCCCCAGAAGAGCGGCGGCTGGACCACTTC
TCTGTCGACGGCTATGACAGTTCTGTAAGGAAGAGCCCAACACTGCTGGAAGTGAGCATGCCCCATTTCATGAGAA

Disease Models & Mechanisms • Supplementary information

CCAACAGCTTCGCCGAGGACCTGGACCTGGAAGGGGAGACTCTGCTGACACCCATCACCCACATCTCACAGCTGCG
GGAACACCATCGGGCCACCATTAAGGTCATTCGACGCATGCAGTACTTTGTGGCCAAGAAGAAATTCTAG

**Kv7.1**$^{Q531X}$ (c.1591C>T)
ATGGCCGCGGCCTCCTCCCCGCCCAGGGCCGAGAGGAAGCGCTGGGGTTGGGGCCGCCTGCCAGGCGCCCGGCGGG
GCAGCGCGGGCCTGGCCAAGAAGTGCCCCTTCTCGCTGGAGCTGGCGGAGGGCGGCCCGGCGGGCGGCGCGCTCTA
CGCGCCCATCGCGCCCGGCGCCCCAGGTCCCGCGCCCCCTGCGTCCCCGGCCGCGCCCGCCGCGCCCCCAGTTGCC
TCCGACCTTGGCCCGCGGCCGCCGGTGAGCCTAGACCCGCGCGTCTCCATCTACAGCACGCGCCGCCCGGTGTTGG
CGCGCACCCACGTCCAGGGCCGCGTCTACAACTTCCTCGAGCGTCCCACCGGCTGGAAATGCTTCGTTTACCACTT
CGCCGTCTTCCTCATCGTCCTGGTCTGCCTCATCTTCAGCGTGCTGTCCACCATCGAGCAGTATGCCGCCCTGGCC
ACGGGGACTCTCTTCTGGATGGAGATCGTGCTGGTGGTGTTCTTCGGGACGGAGTACGTGGTCCGCCTCTGGTCCG
CCGGCTGCCGCAGCAAGTACGTGGGCCTCTGGGGGCGGCTGCGCTTTGCCCGGAAGCCCATTTCCATCATCGACCT
CATCGTGGTCGTGGCCTCCATGGTGGTCCTCTGCGTGGGCTCCAAGGGGCAGGTGTTTGCCACGTCGGCCATCAGG
GGCATCCGCTTCCTGCAGATCCTGAGGATGCTACACGTCGACCGCCAGGGAGGCACCTGGAGGCTCCTGGGCTCCG
TGGTCTTCATCCACCGCCAGGAGCTGATAACCACCCTGTACATCGGCTTCCTGGGCCTCATCTTCTCCTCGTACTT
TGTGTACCTGGCTGAGAAGGACGCGGTGAACGAGTCAGGCCGCGTGGAGTTCGGCAGCTACGCAGATGCGCTGTGG
TGGGGGGTGGTCACAGTCACCACCATCGGCTATGGGGACAAGGTGCCCCAGACGTGGGTCGGGAAGACCATCGCCT
CCTGCTTCTCTGTCTTTGCCATCTCCTTCTTTGCGCTCCCAGCGGGGATTCTTGGCTCGGGGTTTGCCCTGAAGGT
GCAGCAGAAGCAGAGGCAGAAGCACTTCAACCGGCAGATCCCGGCGGCAGCCTCACTCATTCAGACCGCATGGAGG
TGCTATGCTGCCGAGAACCCCGACTCCTCCACCTGGAAGATCTACATCCGGAAGGCCCCCCGGAGCCACACTCTGC
TGTCACCCAGCCCCAAACCCAAGAAGTCTGTGGTGGTAAAGAAAAAAAAGTTCAAGCTGGACAAAGACAATGGGGT
GACTCCTGGAGAGAAGATGCTCACAGTCCCCCATATCACGTGCGACCCCCCAGAAGAGCGGCGGCTGGACCACTTC
TCTGTCGACGGCTATGACAGTTCTGTAAGGAAGAGCCCAACACTGCTGGAAGTGAGCATGCCCCATTTCATGAGAA
CCAACAGCTTCGCCGAGGACCTGGACCTGGAAGGGGAGACTCTGCTGACACCCATCACCCACATCTCACAGCTGCG
GGAACACCATCGGGCCACCATTAAGGTCATTCGACGCATGCAGTACTTTGTGGCCAAGAAGAAATTCCAGTAA

Disease Models & Mechanisms • Supplementary information
